# Generation of a novel, multi-stage, progressive, and transplantable model of plasma cell neoplasms

**DOI:** 10.1038/srep22760

**Published:** 2016-03-10

**Authors:** Takashi Asai, Megan A. Hatlen, Chen Lossos, Delphine Ndiaye-Lobry, Anthony Deblasio, Kazunori Murata, Martin Fleisher, Elena M. Cortizas, Ramiro E. Verdun, John Petrini, Stephen D. Nimer

**Affiliations:** 1 Sylvester Comprehensive Cancer Center, Miller School of Medicine, University of Miami, FL 33136 Miami, USA; 2 Molecular Pharmacology Program, Sloan-Kettering Institute, Memorial Sloan-Kettering Cancer Center, New York, 10065 New York, USA; 3 Department of Laboratory Medicine, Sloan-Kettering Institute, Memorial Sloan-Kettering Cancer Center, New York, 10065 New York, USA; 4 Division of Gerontology and Geriatric Medicine, Department of Medicine, Miller School of Medicine, University of Miami, FL 33136 Miami, USA; 5 Geriatric Research Education and Clinical Center, Miami Veterans Affairs Healthcare System, FL 33125 Miami, USA; 6 Molecular Biology Program, Sloan-Kettering Institute, Memorial Sloan-Kettering Cancer Center, New York, 10065 New York, USA.

## Abstract

Multiple myeloma is a plasma cell neoplasm with an extremely variable clinical course. Animal models are needed to better understand its pathophysiology and for preclinical testing of potential therapeutic agents. Hematopoietic cells expressing the hypermorphic *Rad50*^*s*^ allele show hematopoietic failure, which can be mitigated by the lack of a transcription factor, Mef/Elf4. However, we find that 70% of *Mef*^*−/−*^*Rad50*^*s/s*^ mice die from multiple myeloma or other plasma cell neoplasms. These mice initially show an abnormal plasma cell proliferation and monoclonal protein production, and then develop anemia and a decreased bone mineral density. Tumor cells can be serially transplanted and according to array CGH and whole exome sequencing, the pathogenesis of plasma cell neoplasms in these mice is not linked to activation of a specific oncogene, or inactivation of a specific tumor suppressor. This model recapitulates the systemic manifestations of human plasma cell neoplasms, and implicates cooperativity between the *Rad50*^*s*^ and Mef/Elf4 pathways in initiating myelomagenic mutations that promote plasma cell transformation.

Multiple myeloma is characterized by a slowly progressive monoclonal expansion of plasma cells within the bone marrow, which in most cases is accompanied by a serum monoclonal gammopathy and clinical complications including anemia, multiple bone lesions, nephropathy and frequent infections^1^,^2^. Although outcomes for myeloma patients have greatly improved, multiple myeloma remains an incurable disease, despite the availability of newer treatment modalities^1^.

Several mouse models of multiple myeloma and plasma cell neoplasms have been reported^3^, including xenograft models of human myeloma cells and transplantation models from spontaneously arising or chemically induced murine plasma cell neoplasms^4^,^5^,^6^. In addition, several transgenic mice have been reported to develop multiple myeloma and plasma cell neoplasms^7^,^8^,^9^; these mice were genetically modified to trigger the increased expression of genes, such as *c-Myc*, *XBP-1*, and *MafB*, which have been implicated in the initiation or progression of myeloma in humans. However, these models imperfectly mimic the human disease and while they recapitulate aspects of its clinical and pathogenic features, they are driven by already known genes^10^.

Non-homologous end-joining (NHEJ) pathways, which mainly participate in the repair of DNA double strand breaks (DSBs), contribute to class switch recombination (CSR) in B cells^11^,^12^. The MRN complex, which contains Mre11, Rad50, and Nbs1 in mammalian cells, functions as a sensor of DNA DSBs, regulating DSB repair through homologous recombination and NHEJ pathways, activating ATM as well as ATR signaling^13^. The MRN complex plays multiple roles during CSR in B cells; and is essential for its integrity^14^. The MRN complex also accelerates somatic hypermutation and gene conversion of immunoglobulin variable regions^15^, thereby playing a critical role in humoral immunity mediated by B cells. Mutations in the *Mre11*, *Rad50*, and *Nbs1* genes have been reported in B-cell non-Hodgkin lymphoma and may relate to their pathogenesis^16^,^17^. NHEJ has been implicated in the development of multiple myeloma, with whole genome sequencing of multiple myeloma samples identifying a mutation in the coding region of the *Mre11* gene^18^,^19^, and gene expression profiling of multiple myeloma cells showing increased expression of NHEJ-related genes, such as *Rad50* and *Xrcc4*^20^.

Previously, we reported that hematopoietic cells expressing the hypermorphic *Rad50*^*s*^ allele show constitutive ATM activation, leading to cancer predisposition and progressive hematopoietic failure in *Rad50*^*s/s*^ mice^21^,^22^. In our attempt to mitigate this hematopoietic failure, we crossed *Rad50*^*s/s*^ mice with a variety of mice that lacked cell cycle regulatory genes that may control hematopoietic stem or progenitor cell (HSPC) quiescence and found that the absence of the transcription factor Mef/Elf4 led to the greatest rescue of the hematopoietic failure^23^,^24^,^25^. Based on our previous study, the *Mef*^*−/−*^ *Rad50*^*s/s*^ mice showed lower number of B cells, myeloid cells, NK cells, and HSPCs than wild type controls^25^; however, serendipitously, we observed that many *Mef*^*−/−*^ *Rad50*^*s/s*^ mice died with plasma cell hyperproliferation, which prompted us to generate and more intensively analyze doubly modified mice.

In this study, we have analyzed the phenotypic and genomic abnormalities present in the *Mef*^*−/−*^ *Rad50*^*s/s*^ mice, establishing a novel and transplantable mouse model of multiple myeloma and plasma cell neoplasms which mimics the human disease and is not attributed to the activation of a specific oncogene or inactivation of a specific tumor suppressor gene (other than *Mef*). We have begun to clarify the mechanisms by which the *Mef*^*−/−*^ *Rad50*^*s/s*^ mice develop plasma cell neoplasms. We believe this mouse model will be useful for further analyzing disease initiation and progression, and for further pre-clinical screening of anti-myeloma compounds.

## Results

### Plasma cell neoplasms observed in the *Mef* ^*−/−*^ *Rad50* ^*s/s*^ mice

**Figure 1:**
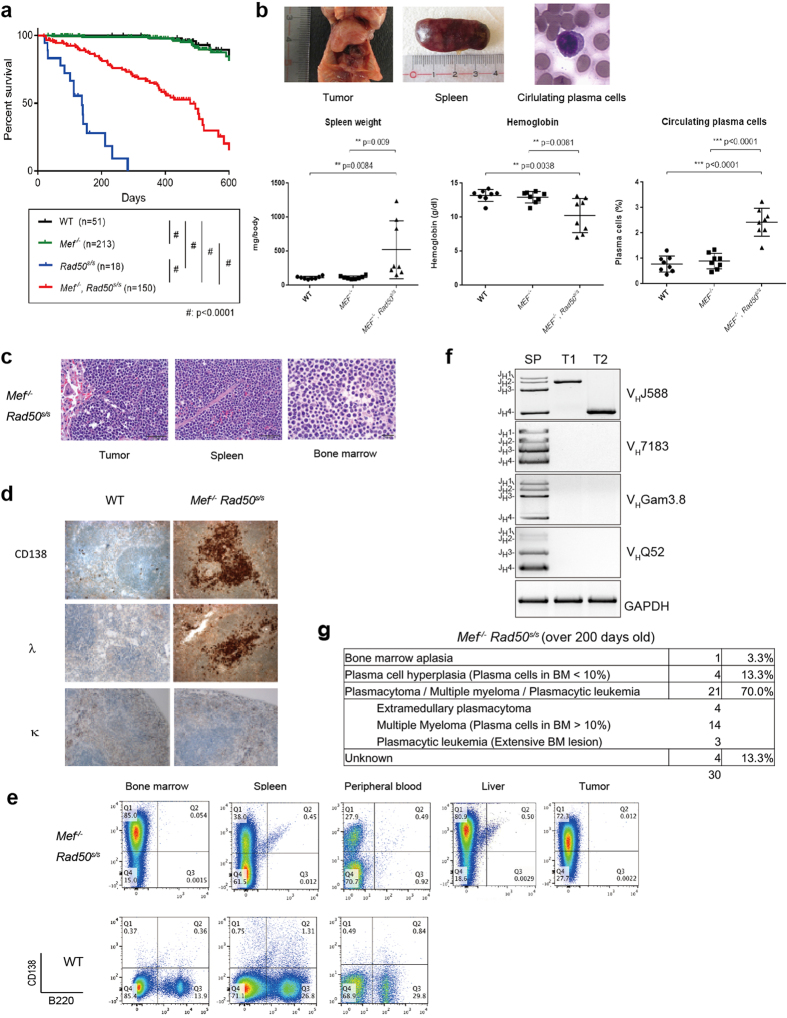
Plasma cell neoplasms observed in the *Mef*^*−/−*^ *Rad50*^*s/s*^ mice. (**a**) Kaplan-Meier curves showing the survival of wild type control, *Mef*^*−/−*^, *Rad50*^*s/s*^, and *Mef*^*−/−*^ *Rad50*^*s/s*^ mice. Mouse number of each group is demonstrated. (**b**) Macroscopic pathological findings of a representative 1-year-old *Mef*^*−/−*^ *Rad50*^*s/s*^ mouse and circulating plasma cells observed in *Mef*^*−/−*^ *Rad50*^*s/s*^ mice in the upper panels. Spleen weight (mg), hemoglobin concentration (g/dL), and frequency of circulating plasma cells in peripheral blood (%) were analyzed in 300–350-day-old wild type, *Mef*^*−/−*^, and *Mef*^*−/−*^ *Rad50*^*s/s*^ mice in the lower panel. P values between wild type and *Mef*^*−/−*^ *Rad50*^*s/s*^ mice, and *Mef*^*−/−*^ and *Mef*^*−/−*^ *Rad50*^*s/s*^ mice are shown in each graph. (**c**) Histological images of tumor, spleen and bone marrow in a representative 1-year-old *Mef*^*−/−*^ *Rad50*^*s/s*^ mouse stained with hematoxylin and eosin at x400 magnification. (**d**) Immunohistochemical staining of the wild type and *Mef*^*−/−*^ *Rad50*^*s/s*^ spleens, using anti-CD138, anti-λ, and anti-κ antibodies. (**e**) Flow cytometric analysis of various tissues from 1-year-old *Mef*^*−/−*^ *Rad50*^*s/s*^ mice and age-matched wild type control mice. The profile of B220 and CD138 expression is shown. The number shows the frequency of each quadrant. (**f**) PCR detection of immunoglobulin gene rearrangements in tumor and normal spleen samples using V_H_ family-specific forward primers and a reverse primer at J_H_4 to amplify variable-joining (V_H_-J_H_) regions of the IgH locus. An independent PCR assay amplifying a region of the GAPDH gene was performed for input control. A clear shift from multi-bands to mono-band can be observed in tumors only on the V_H_J568 amplification, suggesting the monoclonality of the tumor. SP: spleen control from wild type mice; T1, T2: tumor from *Mef*^*−/−*^ *Rad50*^*s/s*^ mice. (**g**) Cause of death in *Mef*^*−/−*^ *Rad50*^*s/s*^ mice, as determined by pathology, immunohistochemistry, and flow cytometry.

We analyzed the phenotypes of the *Mef*^*−/−*^ *Rad50*^*s/s*^ mice with wild type, *Mef*^*−/−*^, and *Rad50*^*s/s*^ mice, and found that *Mef*^*−/−*^ *Rad50*^*s/s*^ mice have a longer survival than *Rad50*^*s/s*^ mice, which is, nonetheless, much shorter than the survival of wild type mice (Fig. 1a). The median survival of the *Mef*^*−/−*^ *Rad50*^*s/s*^ mice was 478 days vs 138 days for the *Rad50*^*s/s*^ mice, and when we examined *Mef*^*−/−*^ *Rad50*^*s/s*^ mice that were greater than 300 days old, many showed severe anemia, increased numbers of plasma cells in the peripheral blood, and/or tumor formation with splenomegaly (Fig. 1b, upper panels). We analyzed 10–12 month-old wild type, *Mef*^*−/−*^, and *Mef*^*−/−*^ *Rad50*^*s/s*^ mice, but not *Rad50*^*s/s*^ mice, which die less than 10 months after birth (Fig. 1a), and found that *Mef*^*−/−*^ *Rad50*^*s/s*^ mice show statistically more anemia, circulating plasma cells, and splenomegaly, compared to the age-matched wild type and *Mef*^*−/−*^ mice (Fig. 1b, lower panels). In addition, extramedullary tumors were observed in 3 of 15 *Mef*^*−/−*^ *Rad50*^*s/s*^ mice 300–500 day-old, but not in 20 age-matched wild type or 20 age-matched *Mef*^*−/−*^ mice, with a significant difference (p = 0.023: wild type vs *Mef*^*−/−*^ *Rad50*^*s/s*^, p = 0.023: *Mef*^*−/−*^ vs *Mef*^*−/−*^ *Rad50*^*s/s*^). Microscopically, we found an extensive infiltration of monotonous plasmacytoid cells throughout the spleen, bone marrow and peripheral blood (Fig. 1c). Immunohistochemical staining of the spleen showed that the plasmocyte-like cells were, in fact, B220 negative, CD138 positive plasma cells (Fig. 1d). We analyzed spleens, bone marrows, and tumors (if available) from 20 wild type, 20 *Mef*^*−/−*^, and 15 *Mef*^*−/−*^ *Rad50*^*s/s*^ mice between the age of 300 and 500 days old by immunohistochemistry. While aggregates of CD138^+^ B220^−^ plasma cells were found in the spleen, bone marrow, and/or tumor in 12 *Mef*^*−/−*^ *Rad50*^*s/s*^ mice (80%), no plasma cell aggregates were observed in wild type or *Mef*^*−/−*^ mice; this too represents a significant abnormality (p < 0.0001: *Mef*^*−/−*^ *Rad50*^*s/s*^ vs wild type, p < 0.0001: *Mef*^*−/−*^ *Rad50*^*s/s*^ vs *Mef*^*−/−*^). Although 95% of mouse B and plasma cells are normally κ light chain positive^26^, 4 of the 18 (22.2%) *Mef*^*−/−*^ *Rad50*^*s/s*^ mice that we analyzed by flow cytometry showed exclusive λ light chain positivity. On the other hand, 14 (77.8%) mice showed κ light chain positivity with λ exclusively negative. These imply an abnormal and likely monoclonal plasma cell proliferation (Supplementary Table S1 and Fig. 1d). When we analyzed cells from various *Mef*^*−/−*^ *Rad50*^*s/s*^ mouse tissues by flow cytometry, we found extensive involvement of multiple organs with CD138^+^ B220^−^ plasma cells (Fig. 1e). These cells did not express cell surface CD3, CD4, or CD8, demonstrating that they are not T cells (data not shown). We also analyzed the immunoglobulin class in each mouse by flow cytometry and found that of the 19 mice analyzed, 16 showed IgG tumors, one mouse showed IgA, and 2 mice had no detectable immunoglobulin heavy chain (Supplementary Table S1).

For the clonality analysis, we performed V(D)J sequencing to examine the clonality of the plasma cell infiltration found in the *Mef*^*−/−*^ *Rad50*^*s/s*^ mice, using PCR to amplify the multiple variable-joining (V_H_-J_H_) regions of the IgH locus. We used the tumors from the *Mef*^*−/−*^ *Rad50*^*s/s*^ mice and the spleen control samples for this analysis, though we were not able to collect enough numbers of purified tumor cells from block samples because of technical limitations. While the PCR products from spleen tissue demonstrated multiple bands, which represent polyclonality, the PCR products from the tumor samples clearly showed a single band, only derived from the V_H_J588 family, representing monoclonality (Fig. 1f). Monoclonal bands from three tumor samples were cloned and sequenced, and we confirmed that all (10 out of 10 sequenced) had the same monoclonal V_H_, D_H_, and J_H_ usage with modest numbers of the same somatic mutations in the V_H_ regions (Table 1). In addition, we performed PCR-based D-J_H_ rearrangement PCR assays on tumor samples from several different *Mef*^*−/−*^ *Rad50*^*s/s*^ mice and found clonal but distinct D-J_H_ rearrangements in the different mice (Supplementary Figure S1). Furthermore, to confirm the clonality of the plasma cells in *Mef*^*−/−*^ *Rad50*^*s/s*^ tumors, we looked for V(D)J rearrangement at the IgH locus by Southern blotting using a J_H_4-Eμ probe and found monoclonal VDJ rearrangement bands only in *Mef*^*−/−*^ *Rad50*^*s/s*^ tumors (Supplementary Figure S2); the control, wild type splenic B cells showed multiple VDJ rearrangement bands. Taken together, these data suggest that the plasma cell infiltrates and tumors in the *Mef*^*−/−*^ *Rad50*^*s/s*^ mice are monoclonal in nature and of post-germinal center origin.

We determined the cause of death in many of the *Mef*^*−/−*^ *Rad50*^*s/s*^ mice (~85%), and found that ~15% of the *Mef*^*−/−*^ *Rad50*^*s/s*^ mice had severe bone marrow failure, while the remaining ~70% developed plasma cell dyscrasias, initially a syndrome resembling monoclonal gammopathy of unknown significance (MGUS), which unlike the human disorder, invariably progressed to multiple myeloma and even plasmacytic leukemia in some mice (Fig. 1g and Supplementary Table S1).

### Progressive plasma cell neoplasms found in the *Mef* ^*−/−*^ *Rad50* ^*s/s*^ mice

**Figure 2:**
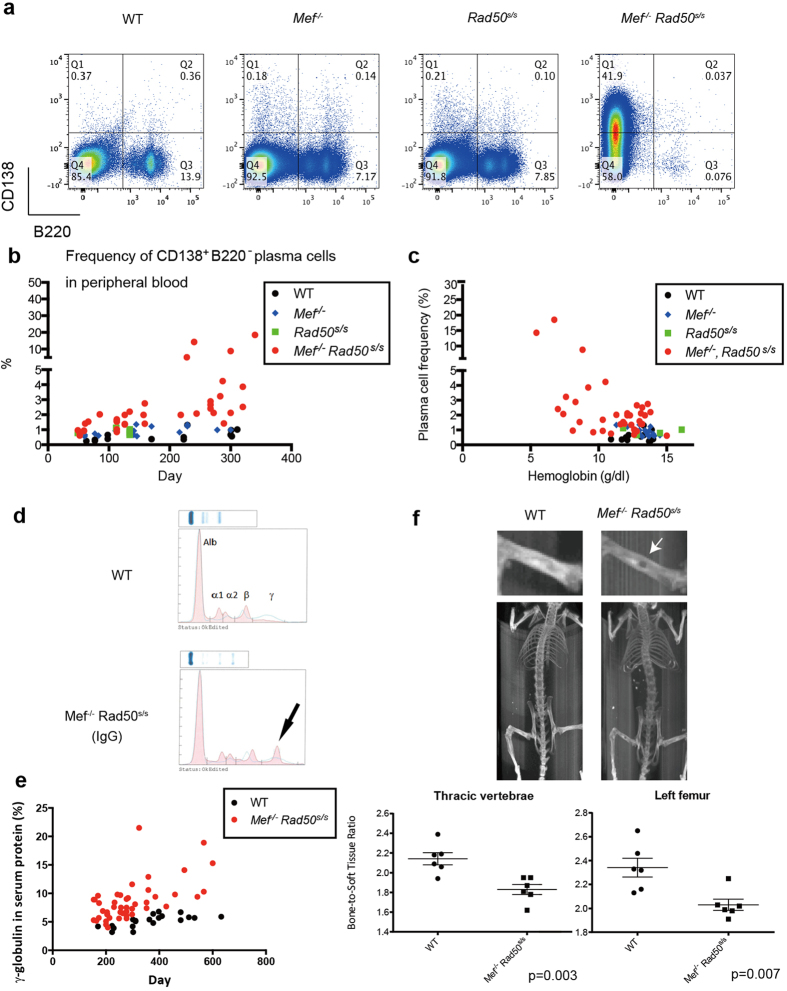
Progressive plasma cell neoplasms found in the *Mef*^*−/−*^ *Rad50*^*s/s*^ mice. (**a**) The B220 and CD138 expression profile of bone marrow cells obtained from 6-month-old wild type control, *Mef*^*−/−*^, *Rad50*^*s/s*^, and *Mef*^*−/−*^ *Rad50*^*s/s*^ mice. The number shows the frequency of cells in each quadrant. (**b**) The frequency of CD138^+^ B220^−^ plasma cells in the peripheral blood is plotted vs the age of the mice. P value is 0.028, comparing the slopes of linear regression by ANCOVA: wild type vs *Mef*^*−/−*^ *Rad50*^*s/s*^ mice. (**c**) The hemoglobin concentration (g/dL) is plotted against the frequency of CD138^+^ B220^−^ plasma cells in the peripheral blood. P value is 0.015, comparing the slopes of linear regression by AVCOVA: wild type vs *Mef*^*−/−*^ *Rad50*^*s/s*^ mice. (**d**) Serum protein electrophoresis of 1-year-old wild type and *Mef*^*−/−*^ *Rad50*^*s/s*^ mice. The M-spike is indicated by the arrow. (**e**) The γ-globulin percentage of total serum protein is plotted vs the age of the corresponding mouse. P value is 0.037, comparing the slopes of linear regression by AVCOVA: wild type vs *Mef*^*−/−*^ *Rad50*^*s/s*^ mice. (**f**) Detection of osteolytic lesions by X-ray analysis of *Mef*^*−/−*^ *Rad50*^*s/s*^ mice (upper panels). The solitary osteolytic lesion is marked by the white arrow. The ratio of the bone density of the thoracic vertebrae and left femur, to the soft tissue density is calculated for the wild type and *Mef*^*−/−*^ *Rad50*^*s/s*^ mice (lower panels) (n = 6 each group). P values are 0.003 (thoracic vertebrae) and 0.007 (left femur), respectively.

We analyzed the frequency of plasma cells in the bone marrow of younger mice (aged 150 to 200 days old) by flow cytometry, and while the bone marrow of the wild type, *Mef*^*−/−*^, *and Rad50*^*s/s*^ mice contained <1% CD138^+^ B220^−^ plasma cells (0.38 ± 0.10%, 0.47 ± 0.26%, and 0.67 ± 0.38%, respectively, n = 4 each), the *Mef*^*−/−*^ *Rad50*^*s/s*^ bone marrows contained significantly larger numbers of plasma cells (14.43 ± 3.41%, n = 4), at a time when the mice showed no signs of disease (Fig. 2a and Supplementary Figure S3). Similarly, the peripheral blood of the *Mef*^*−/−*^ *Rad50*^*s/s*^ mice had higher plasma cell frequencies and more absolute numbers of plasma cells than the other genotypes, with the plasma cell frequency increasing significantly with age (p value is 0.028, comparing the slopes of linear regression by AVCOVA: wild type vs *Mef*^*−/−*^ *Rad50*^*s/s*^ mice) (Fig. 2b). The *Mef*^*−/−*^ *Rad50*^*s/s*^ mice also had lower hemoglobin concentrations than the other mice (aged 200–300 days old, p = 0.0323: *Mef*^*−/−*^ *Rad50*^*s/s*^ vs wild type, p = 0,0156: *Mef*^*−/−*^ *Rad50*^*s/s*^ vs *Mef*^*−/−*^, n = 6 each), and the severity of the anemia significantly correlated with the number of plasma cells in the peripheral blood (p value is 0.015, comparing the slopes of linear regression by AVCOVA: wild type vs *Mef*^*−/−*^ *Rad50*^*s/s*^ mice) (Fig. 2c). We looked for monoclonal protein secretion, and found monoclonal peaks (“M spikes”) on the serum protein electrophoresis of all *Mef*^*−/−*^ *Rad50*^*s/s*^ mice with multiple myeloma or plasmacytic leukemia that were over 300 days old (Fig. 2d and Supplementary Table S1). We also performed serum protein electrophoresis, on younger *Mef*^*−/−*^ *Rad50*^*s/s*^ mice: 12 mice were less than 100 days, 8 were 100–200 days, and 9 were 200–300 days old. These mice were not anemic and they displayed no symptoms; none of the mice less than 100 days old had a monoclonal peak, while two (25%) of the 100–200-day-old mice and four (44%) of the 200–300-day-old mice showed monoclonal peaks. This suggests that clonal plasma cells gradually expand over time and produce more M protein. Similarly, serum γ-globulin levels were also significantly higher in the *Mef*^*−/−*^ *Rad50*^*s/s*^ mice, than the control mice, and γ-globulin levels increased as the mice aged (p value is 0.037, comparing slopes of linear regression by AVCOVA: wild type vs *Mef*^*−/−*^ *Rad50*^*s/s*^ mice) (Fig. 2e). The micro-vessel density in the *Mef*^*−/−*^ *Rad50*^*s/s*^bone marrow was significantly higher than the bone marrow of the wild type controls (p = 0.010) (Supplementary Figure S4).

We looked for the kinds of end organ damage that is observed in human multiple myeloma patients^2^, and analyzed the bone mineral density of the vertebra and femurs of the *Mef*^*−/−*^ *Rad50*^*s/s*^ mice using micro-CT. The micro-CT showed that 4 of the 6 *Mef*^*−/−*^ *Rad50*^*s/s*^mice (over 300 days old) had focal, lytic bone lesions, while none of the 6 wild type littermate controls had lytic lesions. We also calculated the ratio of the bone density to the soft tissue density of the thoracic vertebrae and left femurs in a variety of mice and observed significantly lower bone density in the *Mef*^*−/−*^ *Rad50*^*s/s*^ mice than in the control mice (P = 0.003 and 0.007 for the thoracic vertebrae and the left femur, respectively); thus *Mef*^*−/−*^ *Rad50*^*s/s*^ mice suffer from diffuse osteoporosis (Fig. 2f) and lytic bone lesions, similar to human multiple myeloma patients^27^. We also performed tartrate-resistant acid phosphatase and hematoxylin-eosin staining of the bones to detect osteoclasts in the *Mef*^*−/−*^ *Rad50*^*s/s*^ and wild type mice. The *Mef*^*−/−*^ *Rad50*^*s/s*^ mice showed significantly more tartrate-positive osteoclasts in the femur than did wild type mice (p = 0.013) (Supplementary Figure S5), findings that are also consistent with that seen in human multiple myeloma. We examined the kidneys of *Mef*^*−/−*^ *Rad50*^*s/s*^ mice, by Congo red fluorescence staining and found two of 12 *Mef*^*−/−*^ *Rad50*^*s/s*^ mice with amyloid deposition in their glomerulus, as demonstrated by apple-green birefringence (Supplementary Figure S6). These findings illustrate how *Mef*^*−/−*^ *Rad50*^*s/s*^ mice recapitulate the biological and clinical features of human multiple myeloma and plasma cell neoplasms. We also examined the chemosensitivity of the malignant plasma cells *ex vivo* and found that melphalan inhibited *Mef*^*−/−*^ *Rad50*^*s/s*^ plasma cell proliferation to a greater degree than control plasma cells (Supplementary Figure S7a).

### Transplantability of *Mef* ^*−/−*^ *Rad50* ^*s/s*^ plasma cell neoplasms

**Figure 3:**
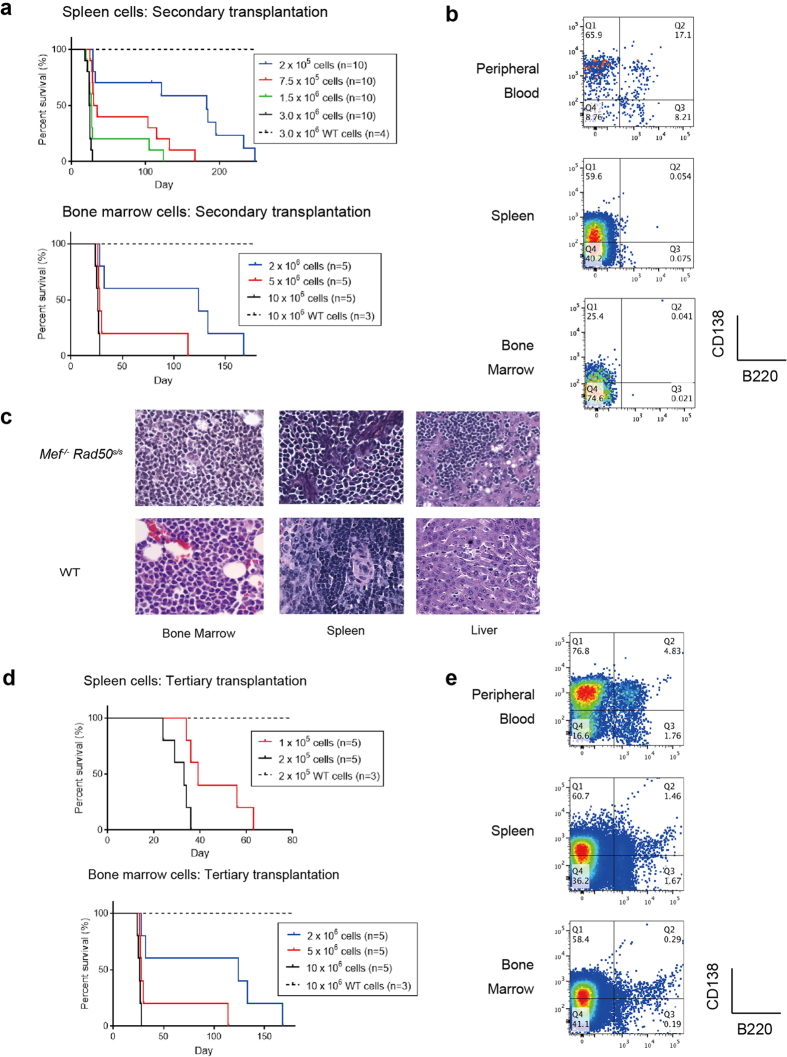
Transplantability of *Mef*^*−/−*^ *Rad50*^*s/s*^ plasma cell neoplasms. (**a**) The Kaplan-Meier curves showing survival after the secondary transplantation of a dose escalating number of spleen and bone marrow cells from tumor-carrying *Mef*^*−/−*^ *Rad50*^*s/s*^ mice or from wild type control mice. (**b**) The profile of B220 and CD138 expression in peripheral blood, spleen and bone marrow cells obtained from recipients 3 weeks after the secondary transplantation of *Mef*^*−/−*^ *Rad50*^*s/s*^ spleen cells. The number shows the frequency of cells in each quadrant. (**c**) The histology of the bone marrow, spleen and liver are shown in recipients 3 weeks after the secondary transplantation of *Mef*^*−/−*^ *Rad50*^*s/s*^ or wild type spleen cells, at x600 magnification. (**d**) The Kaplan-Meier curves showing survival after the tertiary transplantation of a dose escalating number of neoplastic spleen and bone marrow cells or wild type cells from control mice. (**e**) The profile of B220 and CD138 expression in peripheral blood, spleen and bone marrow cells obtained from recipients, 3 weeks after the tertiary transplantation of *Mef*^*−/−*^ *Rad50*^*s/s*^ spleen cells. The number shows the frequency of cells in each quadrant.

We next studied if the plasma cell neoplasms that we observe in *Mef*^*−/−*^ *Rad50*^*s/s*^ mice can be transplanted. Spleen and bone marrow cells from tumor-carrying *Mef*^*−/−*^ *Rad50*^*s/s*^ mice (400–450 days old) were injected into sub-lethally irradiated (4.75Gy) recipient mice in a dose-escalating manner. The recipient mice died within 25–35 days (Fig. 3a). Flow cytometric analysis, and histologic sectioning, demonstrated that the recipient mice suffered from the same plasma cell neoplasms as the original mice (Fig. 3b,c). We measured the bone density in four recipients of 2 × 10^5^ *Mef*^*−/−*^ *Rad50*^*s/s*^ spleen cells and found a modest but significant decrease from 1.908 ± 0.062 to 1.695 ± 0.052 (p = 0.0043), suggesting that the decreased bone density is indeed driven by the plasma cell neoplasm. We also performed tertiary transplantation, using spleen or bone marrow cells collected from 3-week-old recipient mice that had received neoplastic *Mef*^*−/−*^ *Rad50*^*s/s*^ plasma cells. All sub-lethally irradiated recipient mice that received 2 × 10^5^ spleen cells or 1 × 10^5^ bone marrow cells again died within 25–35 days (Fig. 3d), of the same plasma cell disease (Fig. 3e). Fewer cells were needed for the tertiary transplant than the secondary transplant (Fig. 3d), indicating an enrichment for disease-initiating cells with each sequential transplantation.

We also performed *in vivo* treatment of tertiary transplanted mice that had received 2 × 10^5^ spleen cells from the secondary recipients of tumor-carrying *Mef*^*−/−*^ *Rad50*^*s/s*^ mice or age-matched wild type control mice, administered melphalan (2.5 mg/kg, day1–5) or bortezomib (0.5 mg/kg, day 1–4), and following their survival (Supplementary Figure S7b). Both melphalan and bortezomib, which are standard anti-myeloma drugs, significantly prolonged the survival of the mice that had received neoplastic *Mef*^*−/−*^ *Rad50*^*s/s*^ spleen cells, compared to the control vehicle (n = 6, p = 0.0016 and p = 0.0011, respectively). Chesi *et al.* reported that Bortezomib prolonged the survival of secondary or tertiary transplanted Vκ*MYC mice and observed a similar prolongation of survival^28^. Thus, our results of *in vivo* treatment of plasma cell neoplasms is comparable to that obtained using a distinct, transgenic mouse model of multiple myeloma.

### Pathophysiology of *Mef* ^*−/−*^ *Rad50* ^*s/s*^ plasma cell neoplasms

**Figure 4:**
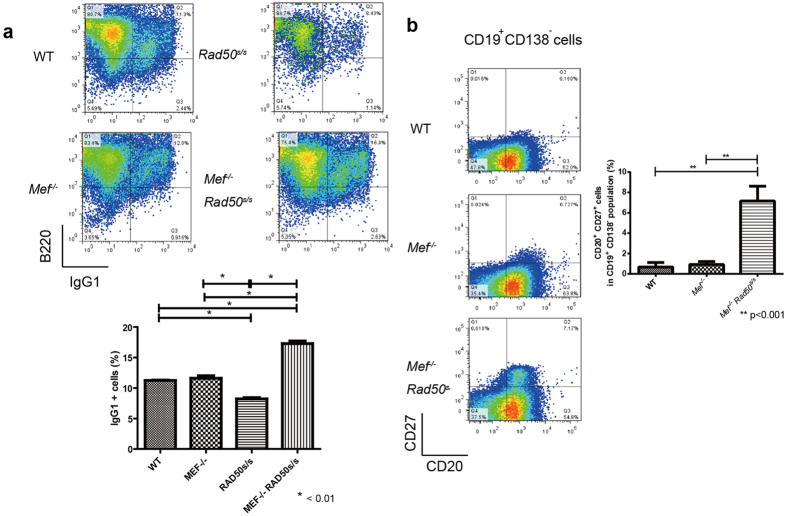
Pathophysiology of *Mef*^*−/−*^ *Rad50*^*s/s*^ plasma cell neoplasms. (**a**) Analysis of *ex vivo* B cell class switch recombination (CSR) of 6-month-old wild type control, *Mef*^*−/−*^, *Rad50*^*s/s*^, and *Mef*^*−/−*^ *Rad50*^*s/s*^ mice. IgG1^+^B cells present after a 96-hour stimulation with anti-CD40 Ab (1 μg/mL) and IL-4 (100 ng/mL), were analyzed by flow cytometry (upper panels). The histogram shows the percentage of stimulated IgG1^+^ B cells from wild type control, *Mef*^*−/−*^, *Rad50*^*s/s*^, and *Mef*^*−/−*^ *Rad50*^*s/s*^ mice (n = 4) (lower panel). (**b**) The profile of CD20 and CD27 expression on the splenic CD19^+^ CD138^−^ B cells of 6-month-old wild type control, *Mef*^*−/−*^, and *Mef*^*−/−*^ *Rad50*^*s/s*^ mice (left panels). The histogram shows the percentage of CD20^+^ CD27^+^ cells in the splenic CD19^+^ CD138^−^ B cells from wild type control, *Mef*^*−/−*^, and *Mef*^*−/−*^ *Rad50*^*s/s*^ mice (n = 4) (right panel).

To address the underlying mechanisms by which the *Mef*^*−/−*^ *Rad50*^*s/s*^ mice develop plasma cell neoplasms, we analyzed the CSR capability of *Mef*^*−/−*^ *Rad50*^*s/s*^ B cells, using flow cytometric analysis of IgG1^+^ B cells after a 4 day *ex vivo* stimulation with anti-CD40 Ab (1 μg/mL) and IL-4 (100 ng/mL). *Mef*^*−/−*^ *Rad50*^*s/s*^ B cells showed more IgG1^+^ cells poststimulation than did the control, *Mef*^*−/−*^, or *Rad50*^*s/s*^ B cells, demonstrating their enhanced CSR capacity (Fig. 4a). We performed V(D)J sequencing of *Mef*^*−/−*^ *Rad50*^*s/s*^ IgG1^+^ “normal” B cells following a 4 day *ex vivo* stimulation with anti-CD40 Ab and IL-4; the PCR products from these cells were polyclonal without specific V(D)J patterns, indicating normal *ex vivo* class switching.

When we phenotypically analyzed the splenic B cells in 200-day-old, apparently healthy control wild type, *Mef*^*−/−*^, *Mef*^*−/−*^ *Rad50*^*s/s*^ mice, we found many more CD20^+^ CD27^+^ CD19^+^ CD138^−^ cells in the *Mef*^*−/−*^ *Rad50*^*s/s*^ spleen (Fig. 4b), cells that are thought to represent post germinal center memory B cells^29^. Mef deficient mice had more splenic B cells than control mice^30^, yet Mef deficiency itself had little impact on CSR or the size of the memory B cell compartment. Thus, Mef deficiency and *Rad50*^*s*^ mutations work synergistically to induce plasma cell transformation.

**Figure 5:**
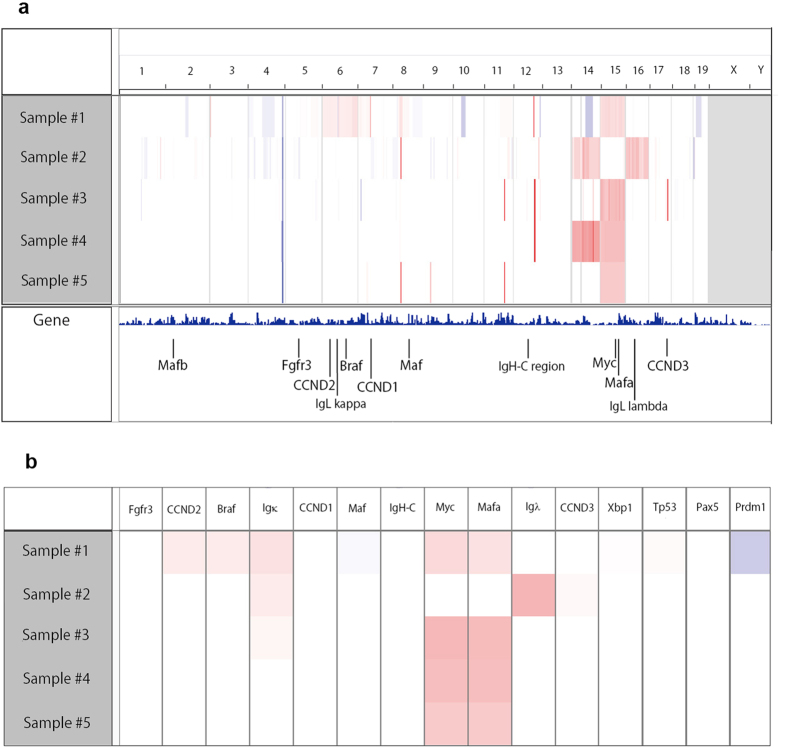
Array CGH of plasma cell neoplasms in *Mef*^*−/−*^ *Rad50*^*s/s*^ mice. (**a**) Array CGH data from 5 different tumor samples in *Mef*^*−/−*^ *Rad50*^*s/s*^ mice. Horizontal marks demonstrate the location of the chromosomes. Red and blue colors mean amplified and decreased locations, respectively. The locations of several myeloma-related genes are indicated below. (**b**) Gene amplification related to human myelomagenesis analyzed from 5 *Mef*^*−/−*^ *Rad50*^*s/s*^ tumor samples by array CGH. Red and blue colors mean amplified and decreased locations, respectively.

We analyzed the level of apoptosis in splenic plasma cells isolated from wild type and *Mef*^*−/−*^ *Rad50*^*s/s*^ 200-day-old mice, and found no difference in the frequency of apoptotic plasma cells (data not shown). We also compared *Bcl2* and *Bax* mRNA expression in wild type plasma cells (n = 4), and *Mef*^*−/−*^ *Rad50*^*s/s*^ plasma cell tumors (n = 8) and found no significant differences, using a qPCR assay (Supplementary Figure S8). Thus, *Mef*^*−/−*^ *Rad50*^*s/s*^ plasma cells have no apparent change in their apoptotic threshold. Amplification, or dysregulated expression, of the c-*Myc* gene is thought to be important for the development of multiple myeloma and plasma cell neoplasms^31^,^32^, so we used array CGH to examine plasma cell samples from five independent *Mef*^*−/−*^ *Rad50*^*s/s*^ mice. Four samples showed high level amplification of chromosome 15, which includes the c-*Myc* gene (Fig. 5a), but we did not find translocation of c-Myc to the Ig locus by FISH analysis (data not shown).

Of the other recurrently translocated genes observed in myeloma patients (*CCND1*, *CCND3*, *MafB*, *Maf*, *Fgfr*, and *Mmset*)^33^, we found *CCND3* amplification in one sample (Fig. 5b). Nonetheless, *CCND3* expression did not vary between wild type plasma cells (n = 4) and the *Mef*^*−/−*^ *Rad50*^*s/s*^ plasma cell tumors (n = 8) using qPCR assays. In contrast, *CCND1* expression was significantly higher in the *Mef*^*−/−*^ *Rad50*^*s/s*^ plasma cell tumors than in wild type plasma cells (Supplementary Figure S9a,b). Increased expression of *CCND* genes is universally observed in MGUS and multiple myeloma, which can disrupt the E2F/RB pathway^10^,^34^. Taken together, these findings mirror the gene expression profiling studies that have compared human plasma cell neoplasm samples and normal human plasma cells^35^,^36^,^37^.

### Myc expression in *Mef* ^*−/−*^ *Rad50* ^*s/s*^ plasma cell neoplasms

**Figure 6:**
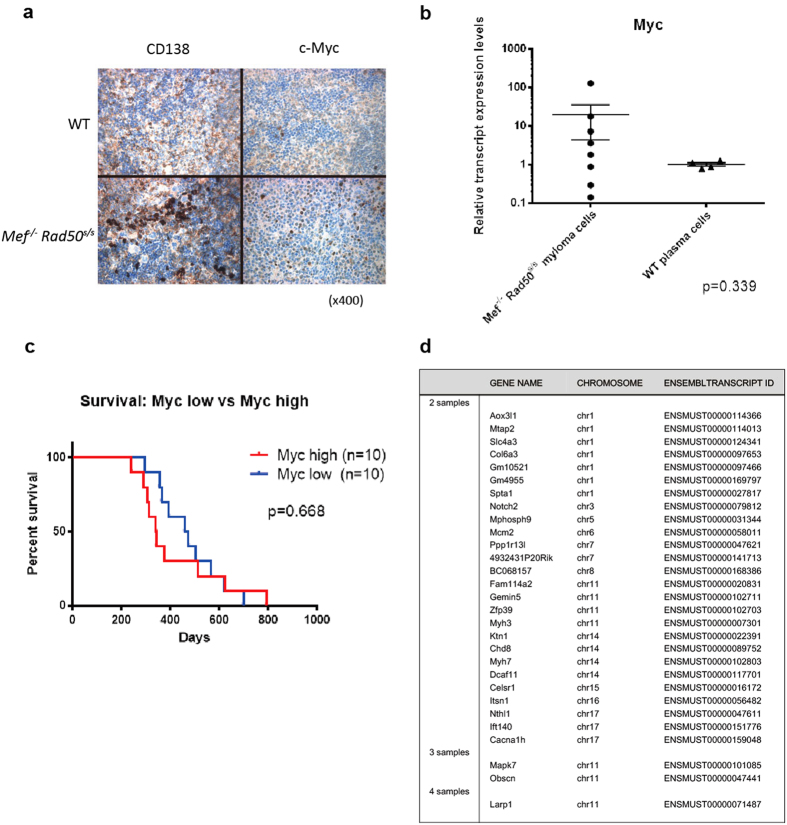
Myc expression in *Mef*^*−/−*^ *Rad50*^*s/s*^ mice. (**a**) Immunohistochemical analysis of WT and *Mef*^*−/−*^ *Rad50*^*s/s*^ spleen sections stained with CD138 and c-Myc, observed at x400 magnification. (**b**) *Myc* transcript expression levels between WT plasma cells (n = 4) vs *Mef*^*−/−*^ *Rad50*^*s/s*^ plasma cell tumors (n = 8) using qPCR. P value is 0.339. (**c**) The survival of two distinct *Mef*^*−/−*^ *Rad50*^*s/s*^ mouse groups, classified by the level of *Myc* transcript expression. The Myc high group has more than twice the Myc transcript level of WT plasma cells, while the Myc low group has less than twice the Myc transcript level of WT plasma cells (n = 10 each group). P value is 0.668 by Log-rank test. (**d**) The result of the exome sequencing for *Mef*^*−/−*^ *Rad50*^*s/s*^ plasma cell tumor samples. List of the somatically mutated genes, found in 2, 3, or 4 different tumor samples. 4 *Mef*^*−/−*^ *Rad50*^*s/s*^ tumor and 5 WT tail samples were used for the exome sequencing.

We also analyzed cell surface CD138 expression and intranuclear Myc expression in the spleen cells of eight affected *Mef*^*−/−*^ *Rad50*^*s/s*^mice (400–450 days old) by immunohistochemistry (Fig. 6a and Supplementary Figure S10). Five samples (62.5%) showed high Myc expression, while three showed no detectable Myc expression, even though CD138 was highly expressed. We also examined *Myc* mRNA expression levels in the *Mef*^*−/−*^ *Rad50*^*s/s*^ plasma cell tumors, using qPCR, and found variable *Myc* expression (both increased and decreased expression) compared to wild type plasma cells (Fig. 6b).

We classified *Mef*^*−/−*^ *Rad50*^*s/s*^ tumor-bearing mice into two groups based on *Myc* mRNA levels, and examined their survival: we found no differences in the survival between the high Myc group (Myc expression 2 x greater than wild type plasma cells) and the low Myc group (where Myc expression was less than 2 x the wild type plasma cells). (Figure 6c). Thus, the *Mef*^*−/−*^ *Rad50*^*s/s*^ intracellular milieu generated plasma cell neoplasms with a variety of *Myc* expression levels, which were comparably aggressive. The plasma cell neoplasms that occur in the *Mef*^*−/−*^ *Rad50*^*s/s*^mice, also mimic what is found in human plasma cell neoplasms, where Myc overexpression is seen in some but not all patients^31^. We also measured *Irf4*, *Prdm1*, and *Xbp-1* expression levels in the *Mef*^*−/−*^ *Rad50*^*s/s*^ plasma cell tumors, and found that the expression of these transcripts was not increased compared to wild type plasma cells (Supplementary Figure S9c–e). This too is similar to what is seen when human plasma cell neoplasms and normal plasma cells are compared^36^,^37^.

### Gene mutations detected by whole exome sequencing found in *Mef* ^*−/−*^ *Rad50* ^*s/s*^ plasma cell neoplasms

We performed whole exome sequencing of four plasma cell tumor samples obtained from *Mef*^*−/−*^ *Rad50*^*s/s*^ mice, using tail samples from five wild-type littermate mice as the germline controls. We found on average 204 exon mutations per sample (range 169–269). One mutated gene (*Larp1*) was seen in all four samples, two mutated genes (*Obscn* and *Mapk7*) were found in 3 of the 4 samples, and 26 mutated genes were found in 2 of the 4 samples (Fig. 6d). These included mutations in the following genes: *Sptn1*, *Mphosph9*, and *Obscn*, all of which have also been observed in human myeloma samples using whole genome sequencing^19^. We validated the mutations in the *Larp1* gene, which were found in 4 samples from *Mef*^*−/−*^ *Rad50*^*s/s*^ mice and the mutations in the *Mapk7* gene, which were found in 3 samples from *Mef*^*−/−*^ *Rad50*^*s/s*^ mice, confirming mutations in *Larp1* for the amino acid changes, A170P, G304W, N593T and S807I, and mutations in *Mapk7* for the amino acid changes, K107R, L167I, V237E, and L368V. Of the 569 genes where we found mutations, 55 genes were identified as having somatic mutations that affect protein-coding regions in human myeloma samples^19^ (Supplementary Table S2). Compared with the currently available genome sequencing data of human multiple myeloma, the plasma cell neoplasms derived from *Mef*^*−/−*^ *Rad50*^*s/s*^ mice possess a wider range of exon mutations, which may imply the absence of one dominant oncogene that drives pathogenesis of the observed plasma cell neoplasms^19^. To identify biologically relevant mutations, we performed functional annotation clustering, using DAVID software, and found ABC transporter, NF-κB signaling, Notch signaling, and focal adhesion signaling clusters to be significantly disturbed (Supplementary Table S3). This suggests that NF-κB signaling pathway genes among others, drive the pathogenesis of *Mef*^*−/−*^ *Rad50*^*s/s*^ driven plasma cell neoplasm, as these genes are also significantly mutated in human multiple myeloma samples^19^.

## Discussion

The *Mef*^*−/−*^ *Rad50*^*s/s*^ mouse is a novel model of human multiple myeloma and plasma cell neoplasms; an abnormal proliferation of plasma cells is seen initially, accompanied by a monoclonal serum protein, mimicking MGUS or smoldering myeloma. However, the mice then develop progressive anemia and osteoporosis, indicative of full-blown myeloma. While MGUS is generally recognized as a premalignant condition that progresses to multiple myeloma at a rate of about 1 percent per year^38^, disease progression in this mouse model occurs with a much greater frequency. In fact, nearly all mice that do not succumb to hematopoietic failure, develop advanced multiple myeloma, or a related plasma cell neoplasm, with time. Thus, this mouse will be useful for studying MGUS and also plasmacytic leukemia, as nearly all of the tertiary transplant recipient mice die of plasmacytic leukemia.

Based on our observations, we hypothesize that the enhanced CSR seen in *Mef*^*−/−*^ *Rad50*^*s/s*^ mice facilitates the accumulation of post-germinal center memory B cells, which together with the genomic instability induced by *Rad50*^*s*^ triggers oncogenic mutations leading to clonogenic plasma cell proliferation^39^. Genomic instability is induced during CSR by activation-induced cytidine deaminase (AID), which has been identified as an enzyme required for somatic hypermutation and CSR^40^. AID has oncogenic activity in post germinal center neoplasms^8^,^41^, thus further studies are needed to clarify whether the development of plasma cell neoplasms in *Mef*^*−/−*^ *Rad50*^*s/s*^ mice is dependent on AID activity. Subsequent mutations, or perhaps epigenetic events, may then trigger the progression to multiple myeloma, or a related plasma cell neoplasm. Clearly, our data suggest that the development of a plasma cell disease in the *Mef*^*−/−*^ *Rad50*^*s/s*^ mice is not necessarily linked to Myc overexpression, which is similar to the situation with human plasma cell neoplasms^35^,^36^,^37^.

The plasma cell neoplasms that we observe in the *Mef*^*−/−*^ *Rad50*^*s/s*^ mice appear to originate from post-germinal center memory B cells, which is consistent with our current understanding of plasma cell biology^42^. Several, recent studies have examined myeloma-initiating cells^39^. For instance, Matsui *et al.* reported that CD20^+^CD27^+^memory B cells isolated from multiple myeloma patients can give rise to clonogenic, multiple myeloma cell growth *in vitro* and engraftment in NOD/SCID mice^29^. The accumulation of post-germinal center, memory B cells likely reflects the earliest steps in the generation of clonogenic plasma cells in the *Mef*^*−/−*^ *Rad50*^*s/s*^ mice. Together with the chromosomal instability that is seen in the *Rad50*^*s/s*^ mouse background, *Mef*^*−/−*^ *Rad50*^*s/s*^ B cells are clearly predisposed to transform into plasma cell malignancies^21^,^25^, likely because they accumulate multiple mutations after CSR.

Our studies of Mef/Elf4, and those of others, have identified both oncogenic and tumor suppressor activities^43^,^44^,^45^,^46^. Perhaps most relevant here are our studies of the role of Mef in the DNA damage response^47^. We found that the absence of Mef diminished the cell’s DNA damage response, leading to less activation of p53 and less γH2AX after irradiation. This could allow for a more modest repair of certain types of DNA damage, and also improved cell survival after cellular stress. *Mef*^*−/−*^ *Rad50*^*s/s*^ mice show a full spectrum of clinical plasma cell disorders, with a time course faster than other mouse models^7^,^8^. One possible reason for this, is that the *Rad50*^*s/s*^ background is more tolerant to the acquisition of mutations. Another is that both *Mef*^*−/−*^ mice^30^ and *Mef*^*−/−*^ *Rad50*^*s/s*^ mice have reduced numbers of NK and NK-T cells (data not shown), which may impair the immune response to the transformed plasma cells that are generated within the *Mef*^*−/−*^ *Rad50*^*s/s*^ mice.

We have summarized the characteristics of the *Mef*^*−/−*^ *Rad50*^*s/s*^ mouse model of multiple myeloma and plasma cell neoplasms, and compared it with other transgenic myeloma models and human multiple myeloma (Supplementary Table S4). Chang *et al.* published that chromosomal instability triggered by Rrm2b loss leads to plasma cell neoplasms^48^. In that model, the malignant cell is provoked by hyperactivation of pro-inflammatory cytokines, including IL-6. IL-6 transgenic mice have been reported to develop extramedullary plasmacytoma^49^. In contrast, neoplastic plasma cells derived from *Mef*^*−/−*^ *Rad50*^*s/s*^ mice do not proliferate *ex vivo* in IL-6 containing media and serum IL-6 levels are not elevated in *Mef*^*−/−*^ *Rad50*^*s/s*^ mice (data not shown). These findings suggest a lack of IL-6 dependence, and indicate a different mechanism of myelomagenesis, than that seen in the Rrm2b null mice.

Interactions between myeloma cells and various components of the bone marrow microenvironment play essential roles in tumor growth, survival, and drug resistance^1^. Increased angiogenesis is thought to be important for the proliferation and survival of myeloma cells, as well as for the disease progression^50^. Published work, using the 5T2MM myeloma mouse model, has demonstrated that multiple myeloma-initiating cells prefer a hypoxic bone marrow microenvironment; nonetheless, hypoxia is apparently lessened during disease progression from MGUS to multiple myeloma^51^. The micro-vessel density is increased in the *Mef*^*−/−*^ *Rad50*^*s/s*^ bone marrow of older mice, which likely supports the progressive growth of the neoplastic plasma cells. Future studies can address the importance of these interactions in our model.

The pathogenesis of the plasma cell neoplasm we observed is not linked to the activation of a specific oncogene nor inactivation of a specific tumor suppressor other than the lack of Mef. Its absence is clearly important because it allows *Rad50*^*s/s*^ mice to survive long enough so they can develop a plasma cell expansion, but its absence clearly plays an important pathogenic role. We do not see increased plasma cells in the circulation or the bone marrow of the *Rad50*^*s/s*^ mice, indicating that the alterations in *Rad50* and *Mef* genes work synergistically to create a cellular environment that promotes plasma cell expansion and transformation. The bone marrow failure induced by *Rad50*^*s/s*^ is partially mitigated by *p21*^*−/*−^, *p27*^*−/−*^, and *Chk2*^*−/−*^ in addition to *Mef*^*−/−*^25, yet *Rad50*^*s/s*^ mice generated on the *p21*^*−/*−^, *p27*^*−/−*^, or *Chk2*^*−/−*^ background primarily develop lymphomas (Morales M *et al.* unpublished data), while the *Rad50*^*s/s*^ mice generated on the *Mef*^*−/−*^background develop only plasma cell neoplasms. Thus, while the *Rad50* hypermorphic status has strong oncogenic potential in the hematopoietic compartment, both *Rad50* hypermorphic status and *Mef* deletion are needed to develop plasma cell neoplasms. Further, in-depth examination of *Mef*^*−/−*^ *Rad50*^*s/s*^ plasma cells will allow us to better define myeloma pathogenesis and screen for novel anti-myeloma compounds, or for factors that can delay the onset or progression of plasma cell disorders.

## Methods

### Mice

The generation of *Mef*^*−/−*^ and *Rad50*^*s/s*^ mice that were backcrossed to C57BL/6 five times was described previously^21^,^30^. All mice were maintained in the Memorial Sloan-Kettering Cancer Center (MSKCC) and University of Miami (UM) Animal Facility, according to IACUC (Institutional Animal Care and Use Committee)-approved protocols, and kept in Thorensten units with filtered germ-free air. All the studies were approved by IACUC of MSKCC and UM and experiments were conducted in accordance with the committee’s approved guidelines.

### Pathological and immunohistochemical studies

Peripheral blood was collected from tail veins and analyzed on an automated blood counter, HEMAVET HV950FS (Drew Scientific). Tissue samples were fixed immediately after isolation and processed into paraffin, sectioned and examined histologically using hematoxylin and eosin, Congo red, or immunohistochemical techniques. Immunohistochemical staining was performed using the following anti-mouse antibodies: CD138 (281-2, BD Pharmingen), λ (SouthernBiotech), κ (SouthernBiotech), and c-Myc (Y-69, Abcam). Samples were reviewed by pathologists and diagnosed using uniform criteria.

### Transplantation studies

Femoral and tibial bone marrow or splenic nucleated cells from the 400–450-day-old *Mef*^*−/−*^ *Rad50*^*s/s*^ mice with plasma cell neoplasms were injected intravenously into sub-lethally (4.75 Gy) irradiated 6–8 week-old C57BL/6 SJL (Jackson Lab) recipient mice.

### Flow cytometry

The following antibodies were used in combinations: CD138, CD45R/B220, CD20, CD27, IgG1, κ and λ (BD Pharmingen). To detect intracellular κ and λ, we used an Intracellular Staining Kit (Invitrogen). Stained cells were analyzed by flow cytometry using FACScan, FACSCalibur (BD), MoFlo (Cytomation) or LSRII (BD).

### Serum protein electrophoresis

Serum protein electrophoresis was performed using a SPIFE 3000 electrophoresis analyzer (Helena Laboratories).

### Clonality and V_H_ analysis

In order to perform the clonality analysis, we examined the tumor and the spleen control samples, though we were not able to collect enough numbers of purified tumor cells from tumor block samples because of technical limitations. We used PCR to amplify the variable-joining (V_H_-J_H_) region of the immunoglobulin heavy chain locus. For this goal the 5′ primers for V_H_J558 (5′-RGCCTGGGRCTTCAGTGAAG-3′ or 5′-AAGGSCACAYTKACTGTAGAC-3′)^52^,^53^ (R = A^+^ G), V_H_GAM3.8: (5′-GAAGAA GCCTGGAGAGACAGTCAAGAT-3′), V_H_Q52: (5′ GCCCTCACAGAGCCTGTCCAT-3′), and V_H_7183: (5′TCCCTGAAACTCTCCTGTGCAGCCTC-3′) were combined with a 3′ primer for J4 (5′-GGAGACGGTGACTGAGGTTCC-3′) were combined with a 3′ primer for J4 (5′- GGAGACGGTGACTGAGGTTCC-3′). Each PCR reaction had a final volume of 25 μl containing 30ng genomic DNA (tumor or spleen), 1 μM of V_H_J558, V_H_GAM3.8, V_H_Q52, or V_H_7183 primer and 0.22 μM of J4 primer. All amplifications were performed with AmpliTaq Gold (Applied Biosystems) with a 10 minute initial denaturation step at 95 ^o^C followed by 11 cycles with 30 second denaturation (94 ^o^C), 1 minute annealing (68 ^o^C, −1 ^o^C per cycle), and 1 minute extension (68 ^o^C); and finally 30 cycles with 30 second denaturation (94 ^o^C), 1 minute annealing (57 ^o^C), and 1 minute extension (68 ^o^C). The PCR products were analyzed in 1.5% Agarose gels with ethidium bromide and cloned into the pCR 2.1 vectors (Invitrogen) for DNA sequencing analysis. Primers used to amplify GAPDH gene were FW (5′- CACCTTCAGCTTTCCGGCCACTTAC-3′) and RV (5′- GGAAGCCCATCACCATCTTCCAGGA-3′). Sequences were performed by 3130xL Genetic Analyzer (Applied Biosystems) and analyzed using MacVector software, and the V_H_, D_H_, and J_H_ usage and mutations were scored by comparing each sequence (10 sequences per sample) with the germline sequences at the IMGT server (http://www.imgt.org)^54^. Based on the nucleic acid numbers of the rearranged V_H_ regions, we calculated the percentage of mutations, as described previously^8^.

### Micro-CT images and measurement of bone mineral density

Mice over 300 days old age were used for this analysis. CT images were obtained using a microCAT-II scanner (ImTek, Knoxville, TN). The image data were processed by an ultra-fast volume reconstruction engine (Real-time Image Reconstruction System), as previously described^55^.

### Array CGH

Genomic DNA was extracted from tissues by DNeasy Tissue & Blood Kit (Qiagen) and the SurePrint G3 Mouse CGH Microarray Kit, 1 × 1M (Agilent Technologies) was used for array CGH analysis. The acquired data were normalized using the MSKCC software and analyzed using the Integrated Genomics Viewer software.

### Quantitative PCR

Quantitative PCR was performed by 7500 Fast Real-Time PCR System (Applied Biosystems), using RNA isolated from wild type plasma cells, which were sorted by mouse CD138^+^ Plasma Cell Isolation Kit (Miltenyi Biotec), and plasma cell tumors derived from *Mef*^*−/−*^ *Rad50*^*s/s*^ mice. Transcript expression levels were calculated and standardized by the ratio of each transcript vs Hprt. The following Taqman probes (Life Technologies) were used for quantitative PCR: Mm00487804_m1 (Myc).

### Exome Sequencing

SureSelect Mouse All Exon Kit (Agilent Technologies) was used for enrichment of the entire mouse exome, and the 5500xl Genetic Analyzer (Applied Biosystems) was used for the sequencing. The BAM files were processed using the GATK toolkit, following the published best practice guidelines. They were first realigned using the InDel realigner and then the base quality values were recalibrated using the BaseQRecalibrator. Variants were then called using the GATK Unified Genotyper. The calls were filtered to remove any mutations scored as LowQual by the Unified Genotyper or with an alternative allele depth <5 reads. The filtered calls were annotated with SNPEff and synonymous mutations were also filtered out from the list. To make the final list from this list, we selected genes with >0.15 of the variant frequency and variants which could not be observed in control samples, and excluded identical variants at an identical base as artifacts.

### Statistics

Statistical significance was assayed by Student’s t test (for two groups) and one-way ANOVA with Tukey’s multiple comparison test as a post test (for more than two groups). Survival analysis was performed by Log-rank test. Comparison of slopes of linear regression is performed by ANCOVA; *p < 0.05; **p < 0.01; ***p < 0.005; # < 0.0001; ns, not significant.

## Additional Information

**How to cite this article**: Asai, T. *et al.* Generation of a novel, multi-stage, progressive, and transplantable model of plasma cell neoplasms. *Sci. Rep.* **6**, 22760; doi: 10.1038/srep22760 (2016).

## Supplementary Material

Supplementary Information

## Figures and Tables

**Table 1:** Mutation analysis of VDJ region in *Mef*^*−/−*^ *Rad50*^*s/s*^ plasma cell tumor samples.

Mice UID	Tumor	V_H_	D_H_	J_H_	No.Mutationsat V_H_	Mutations atV_H_ andAmion Acid Changes	Percent Mutationsat V_H_
33	MM	IGHV1-50^*^01, IGHV1-59^*^01 or IGHV1S40^*^01	IGHD2-3^*^01	IGHJ4^*^01	1	c227 > g, A76 > G	1.0
112	MM	IGHV1-67^*^01	IGHD4-1^*^01	IGHJ3^*^01	2	c227 > g, A76 > G a232 > t, M78 > L	2.1
217	PCT	IGHV1-67^*^01	IGHD3-2^*^02	IGHJ3^*^01	7	c227 > g, A76 > G	7.3
						a232 > t, M78 > L	
						g25 > c, S85 > T	
						a256 > t, T86 > S	
						a263 > t, Y88 > F	
						a290 > g, E97 > G	
						g291 > a, E97 > G	

The first two columns show mouse UID and the phenotype of the mice (MM, multiple myeloma; PCT, solitary plasmacytoma). The V_H_, D_H_, and J_H_ usage and mutations were scored by comparing each sequence with the germline sequences at the IMGT server.
