# Supplementary material for: Generation of a novel, multi-stage, progressive, and transplantable model of plasma cell neoplasms
Source: Sci Rep. 2016 Mar 10;6:22760. doi: 10.1038/srep22760 (PMC4785351; doi:10.1038/srep22760)
Supplement: Supplementary Information [file srep22760-s1.pdf]

Supplementary Information

Generation of a novel, multi-stage, progressive, and transplantable model of plasma cell neoplasms

Takashi Asai, Megan A. Hatlen, Chen Lossos, Delphine Ndiaye-Lobry, Anthony Deblasio, Kazunori Murata, Martin Fleisher, Elena M Cortizas, Ramiro E Verdun, John Petrini, Stephen D Nimer

Supplementary Methods

Southern Blot analysis of V(D)J rearrangement

DNA from wild-type splenic B-cells as well as tumor cells was isolated with proteinase K treatment followed by phenol extraction and ethanol precipitation. Purified DNA was digested with EcoRI and analyzed by Southern blot with a ^{32}P-labeled J_H4-Eμ probe as described previously (Ref 1). The primer sets used for the 1858bp J_H4-Eμ probe are: J4-Eμ FW 5'-CCTCAGCTCCCATACTTCATGGCCA3'; and J4-Emu RV 5'-CTCAGCCTGGACTTTCGGTT-3'.

Quantitative PCR

Quantitative PCR was performed by 7500 Fast Real-Time PCR System (Applied Biosystems), using RNA isolated from wild type plasma cells, which were sorted by mouse CD138+ Plasma Cell Isolation Kit (Miltenyi Biotec), and plasma cell tumors derived from *Mef*$^{-/-}$ *Rad50*$^{s/s}$ mice. Transcript expression levels were calculated and standardized by the ratio of each transcript vs Hprt. The following Taqman probes (Life Technologies) were used for quantitative PCR: Mm00432050_m1 (Bax),

Mm02528810_s1 (Bcl), Mm00432359_M1 (Ccnd1), Mm01612362_m1 (Ccnd3), Mm00446968_m1 (Hprt), Mm00516431_m1 (Irf4), Mm00487804_m1 (Myc), Mm00446968_m1 (Prdm1), and Mm00457357_m1 (Xbp-1).

Measurement of micro-vessel densities

Micro-vessel densities were measured, by the method described previously (Ref. 2).

Tartrate resistant acid phosphatase staining

Tartrate resistant acid phosphatase staining was performed using Acid Phosphatase, Leukocyte (TRAP) Kit (Sigma-Aldrich 387A).

Drug inhibitory test

Drug inhibitory test was performed ex vivo after 48 hours of drug administration, using the WST-8 kit (Cell Counting Kit-8, Dojindo).

***In vivo* treatment for plasma cell neoplasms**

$2x10^5$ spleen cells from the secondary recipients of tumor-carrying *Mef*$^{-/-}$ *Rad50*$^{s/s}$ and age-matched wild type, control mice were transplanted into sub-lethally irradiated

recipient mice. Subsequently, melphalan (2.5mg/kg, day1-5) or bortezomib (0.5mg/kg, day 1-4) was administered to these mice. The survival of these mice was observed.

Exome Sequencing

SureSelect Mouse All Exon Kit (Agilent Technologies) was used for enrichment of the entire mouse exome, and the 5500xl Genetic Analyzer (Applied Biosystems) was used for the sequencing. The data processing pipeline for detecting variants was as follows: First, the FASTQ files were processed to remove any adapter sequences at the end of the reads and trimmed to remove bases with a Q<3. Reads shorter then 50bp were discarded and the files repaired to account for dropped reads. The clipped/trimmed reads were mapped and paired using the BWA mapper: bwa aln and bwa sampe or samse for unpaired reads. The mapped reads were processed with the Picard tools to sort by coordinate (SortSam) and have duplicated reads removed (MarkDuplicates). The BAM files were then processed using the GATK toolkit, following the published best practice guidelines. They were first realigned using the InDel realigner and then the base quality values were recalibrated using the BaseQRecalibrator. Variants were then called using the GATK Unified Genotyper. The calls were filtered to remove any mutations scored as LowQual by the Unified Genotyper or with an alternative allele

depth < 5 reads. The filtered calls were annotated with SNPEff and synonymous mutations were also filtered out from the list. To make the final list from this list, we selected genes with >0.15 of the variant frequency and variants which could not be observed in control samples, and excluded identical variants at an identical base as artifacts.

Ref 1. J Immunol. 191(11):5751-63, 2013. doi: 10.4049/jimmunol.1301300.

Ref. 2 Ann Hematol. 79:574-577, 2000

Supplementary Figure and Table Legends

Supplementary Figure S1: The results of D-JH rearrangement analysis, detected by PCR.

Rearrangement of D to J_1, J_2, J_3, or J_4 is indicated by arrowheads. Plasmacytoma samples from 2 different mice are shown as #1 and #2. The wild type spleen samples show 4 different D-J_H rearrangement bands, while the different tumor samples derived from *Mef*$^{-/-}$ *Rad50*$^{s/s}$ mice show different monoclonal bands.

Supplementary Figure S2: V(D)J Southern blotting of control and *Mef*$^{-/-}$ *Rad50*$^{s/s}$ tumor sample.

DNA of the indicated tumor and control spleen was digested with EcoRI and analyzed for V(D)J rearrangements at the IgH locus by Southern blotting using a 3’ J_H4 probe. Wild-type splenic B cells were used as a positive control to detect IgH rearrangements (indicated by arrowheads).

Supplementary Figure S3: Percentage of CD138$^+$ B220$^-$ plasma cells in bone marrow.

Bone marrows were obtained from 6-month-old wild type control, *Mef*$^{-/-}$, *Rad50*$^{s/s}$, and *Mef*$^{-/-}$ *Rad50*$^{s/s}$ mice (n=4 each). Data are calculated from FACS data. P values are 0.0002 (wild type vs *Mef*$^{-/-}$ *Rad50*$^{s/s}$), 0.0002 (*Mef*$^{-/-}$ vs *Mef*$^{-/-}$ *Rad50*$^{s/s}$), and 0.0002 (*Rad50*$^{s/s}$ vs *Mef*$^{-/-}$ *Rad50*$^{s/s}$), respectively.

Supplementary Figure S4: Micro-vessel density measurement in bone marrow.

Micro-vessel densities were measured on 8 *Mef*$^{-/-}$ *Rad50*$^{s/s}$, 4 *Mef*$^{-/-}$ and 4 wild type bone marrow sections. All these mice were derived from the mice over 300 days old. P values are 0.010 (wild type vs *Mef*$^{-/-}$ *Rad50*$^{s/s}$) and 0.018 (*Mef*$^{-/-}$ vs *Mef*$^{-/-}$ *Rad50*$^{s/s}$), respectively.

Supplementary Figure S5: The number of tartrate-positive osteoclasts per square millimeter in femur sections.

Tartrate-resistant acid phosphatase and hematoxylin-eosin staining of the bones were performed to detect osteoclasts in the *Mef*$^{-/-}$ *Rad50*$^{s/s}$ mice (n=4) compared to wild type mice (n=4) and *Mef*$^{-/-}$ mice (n=4). The numbers of tartrate-positive osteoclasts in femurs

were counted under microscopy and the numbers per square millimeter were calculated. The *Mef*$^{-/-}$ *Rad50*$^{s/s}$ mice showed significantly more osteoclasts in femurs than wild type and *Mef*$^{-/-}$ mice. P values are 0.0013 and 0.0009, respectively by t-test.

Supplementary Figure S6: Amyloid deposits in *Mef*$^{-/-}$ *Rad50*$^{s/s}$ kidneys detected by Congo red staining.

Microscopic pathological findings of the kidneys of 1-year-old *Mef*$^{-/-}$ *Rad50*$^{s/s}$ mice stained with Congo red, and viewed using a fluorescent microscope (x200).

Supplementary Figure S7: *Ex vivo* and *in vivo* cytotoxicity test of anti-myeloma drugs to *Mef*$^{-/-}$ *Rad50*$^{s/s}$ plasma cell neoplasms.

(a) Plasma cell tumor cell lines from *Mef*$^{-/-}$ *Rad50*$^{s/s}$ plasma cells and wild type plasma cells were analyzed for cytotoxicity to melphalan using the WST-8 kit (Cell Counting Kit-8). (b) *In vivo* treatment of *Mef*$^{-/-}$ *Rad50*$^{s/s}$ plasma cell neoplasms using a tertiary transplantation model. 2x10^{5} spleen cells from the secondary recipients of tumor-carrying *Mef*$^{-/-}$ *Rad50*$^{s/s}$ or age-matched wild type control mice were transplanted into sub-lethally irradiated recipient mice. Subsequently, melphalan (2.5mg/kg, day1-5) or bortezomib (0.5mg/kg, day 1-4), or vehicle control was administered to these mice.

The Kaplan-Meier curves show survival after the transplantation and drug administration. Both melphalan and bortezomib significantly prolonged the survival of the recipient mice that had received neoplastic *Mef*$^{-/-}$ *Rad50*$^{s/s}$ spleen cells. P values and sample numbers (n) are presented in the figures.

Supplementary Figure S8: Analysis of transcript expression levels related to apoptosis.

Bcl2 and *Bax* transcript expression levels in *Mef*$^{-/-}$ *Rad50*$^{s/s}$ plasma cell tumors (n=8), wild type plasma cells (n=4), *Mef*$^{-/-}$ plasma cells (n=4), and *Rad50*$^{s/s}$ plasma cells (n=4), measured by qPCR.

Supplementary Figure S9: Transcript expression level of some genes related to myelomagenesis.

(a) *Ccnd1*, (b) *Ccnd3*, (c) *Prdm1*, (d) *Xbp1*, and (e) *Irf4* transcript expression levels in *Mef*$^{-/-}$ *Rad50*$^{s/s}$ plasma cell tumors (n=8), wild type plasma cells (n=4), *Mef*$^{-/-}$ plasma cells (n=4), *Rad50*$^{s/s}$ plasma cells (n=4), and wild type splenic B cells (n=4), measuring by qPCR.

Supplementary Figure S10: Immunohistochemical analysis of Myc and CD138 in *Mef*$^{-/-}$ *Rad50*$^{s/s}$ tumors.

Immunohistochemical analysis of WT and 8 different *Mef*$^{-/-}$ *Rad50*$^{s/s}$ spleen sections stained by CD138 and c-Myc. All slides were observed under the blight field microscope with x400 magnification.

Supplementary Table S1: List of the *Mef*$^{-/-}$ *Rad50*$^{s/s}$ mice died over 200 days old.

The mouse UID, sex, and survival day are listed with the results of pathological and FACS analyses which we performed on the day mice died. The table also demonstrates κ / λ deviation observed by FACS analysis and the existence of monoclonal peaks detected by serum protein electrophoresis.

Supplementary Table S2: List of the somatically mutated genes and mutations, which were identified in the list of somatic mutations observed in human multiple myeloma patients by the whole genome sequencing.

4 *Mef*$^{-/-}$ *Rad50*$^{s/s}$ tumor and 5 WT tail samples were used for this Exome sequencing.

Supplementary Table S3: Functional Annotation Clustering by DAVID Software using the gene mutations which we found in *Mef$^{-/-}$ Rad50$^{s/s}$* plasma cell neoplasms.

4 *Mef$^{-/-}$ Rad50$^{s/s}$* tumor and 5 WT tail samples were used for this Exome sequencing. Only statistically significant ($p<0.05$) clustering are listed.

Supplementary Table S4: Comparison of the biological and clinical features of multiple myeloma mouse models.

Supplementary Figure S1

The results of D-JH rearrangement analysis. detected by PCR.

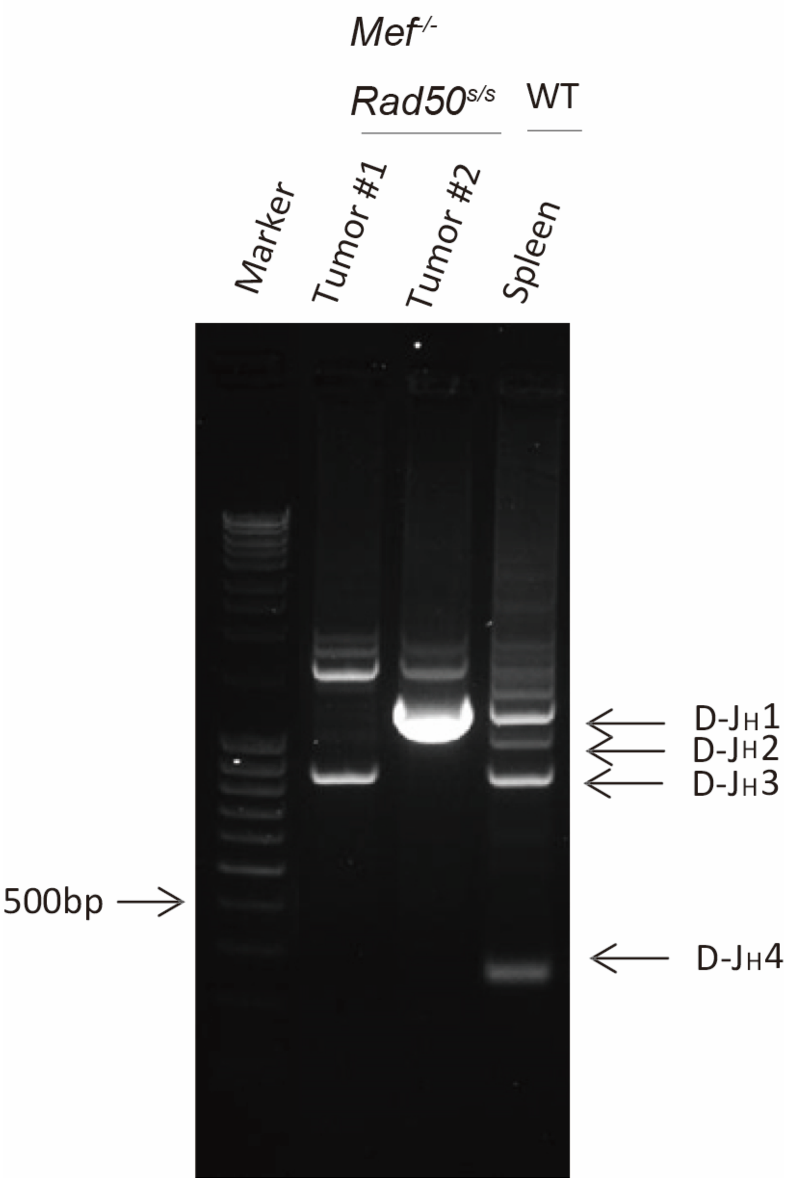

Supplementary Figure S2

V(D)J Southern blotting of control and *Mef*$^{-/-}$ *Rad50*$^{s/s}$ tumor sample.

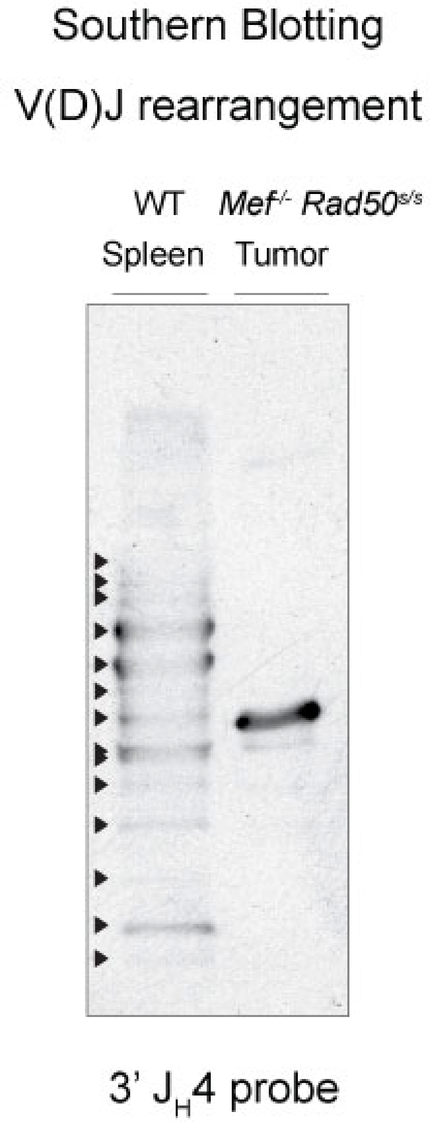

Supplementary Figure S3

Percentage of $CD138^+$ $B220^-$ plasma cells in bone marrow.

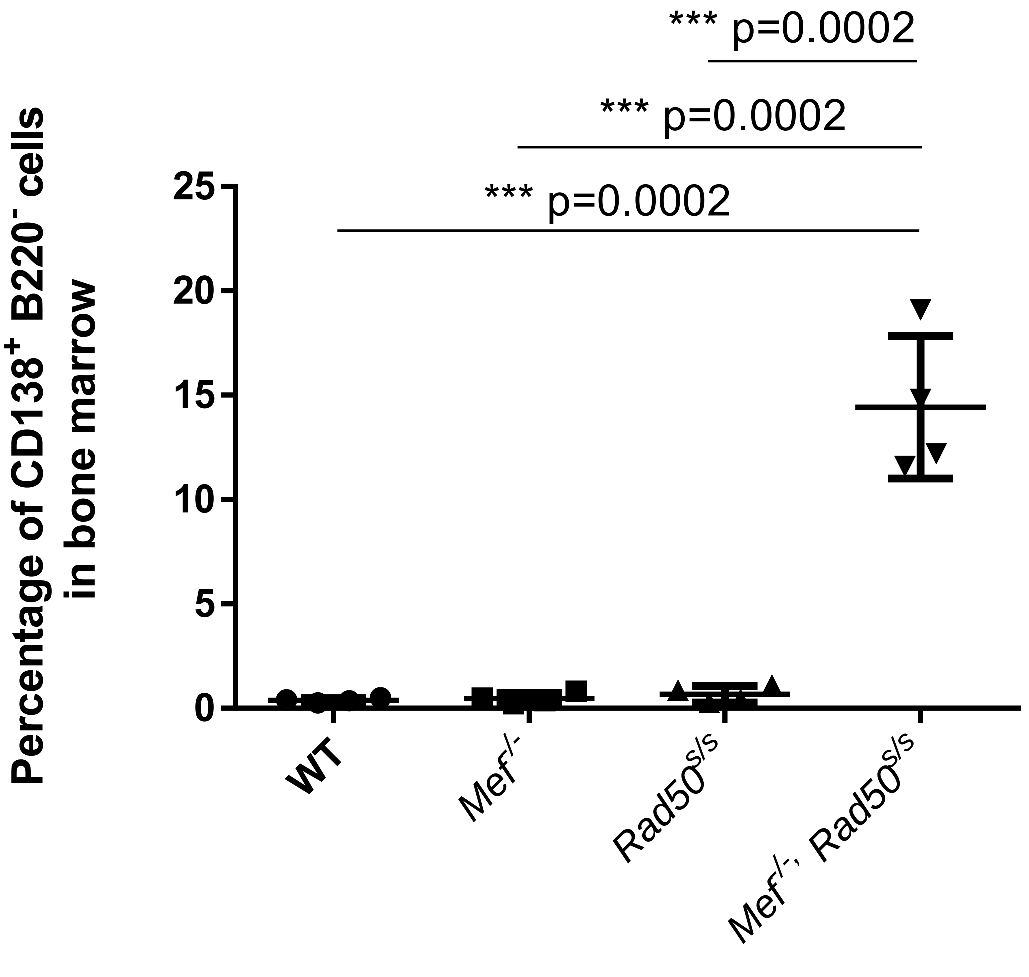

Supplementary Figure S4

Micro-vessel density measurement in bone marrow sections.

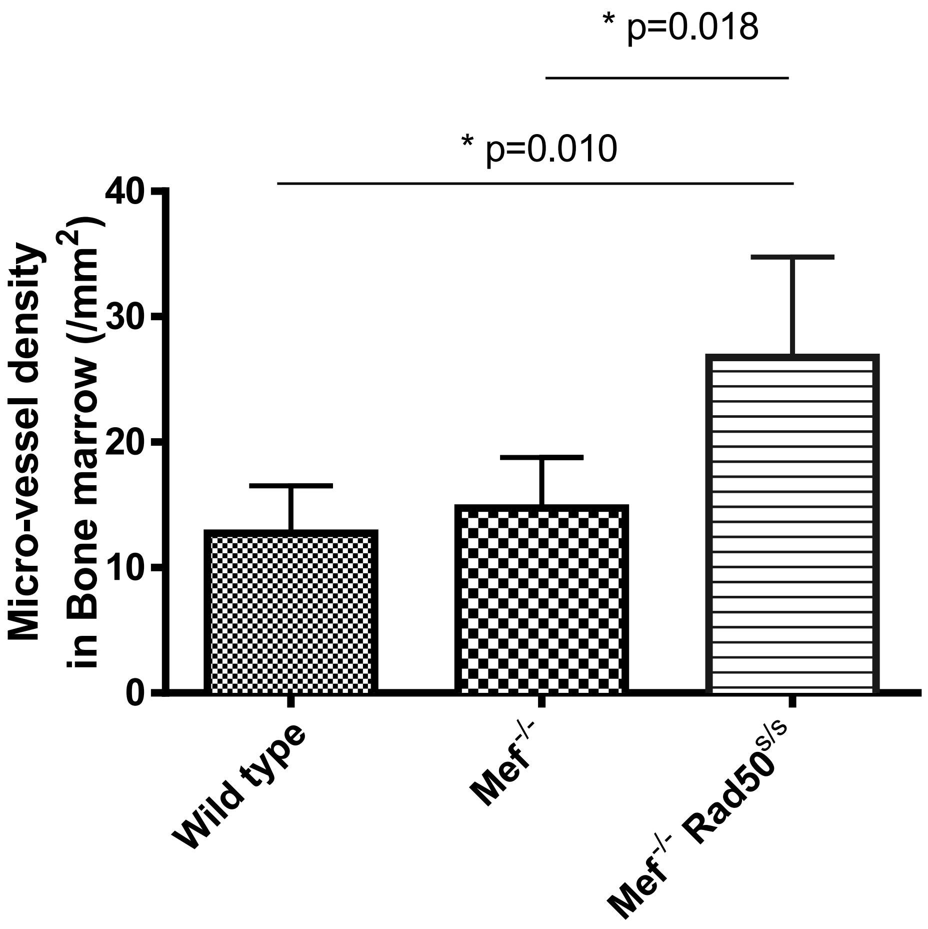

Supplementary Figure S5

The number of tartrate-positive osteoclasts in femurs per square millimeter.

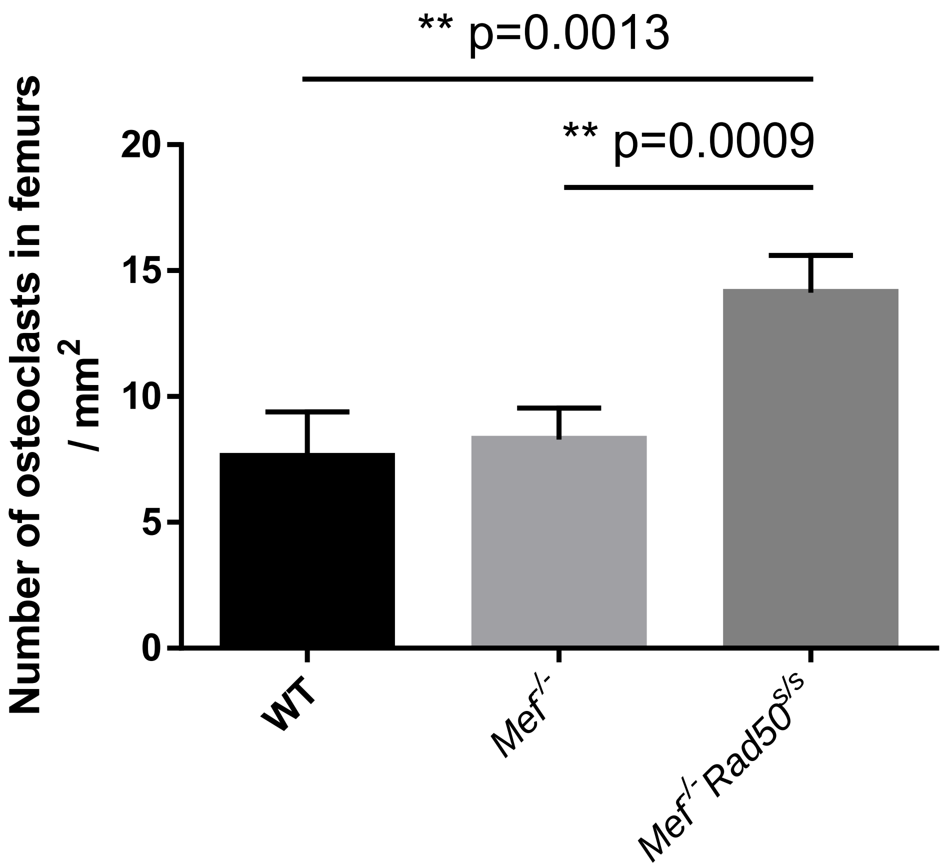

Supplementary Figure S6

Amyloid deposits in *Mef*$^{-/-}$ *Rad50*$^{s/s}$ kidneys detected by Congo red staining.

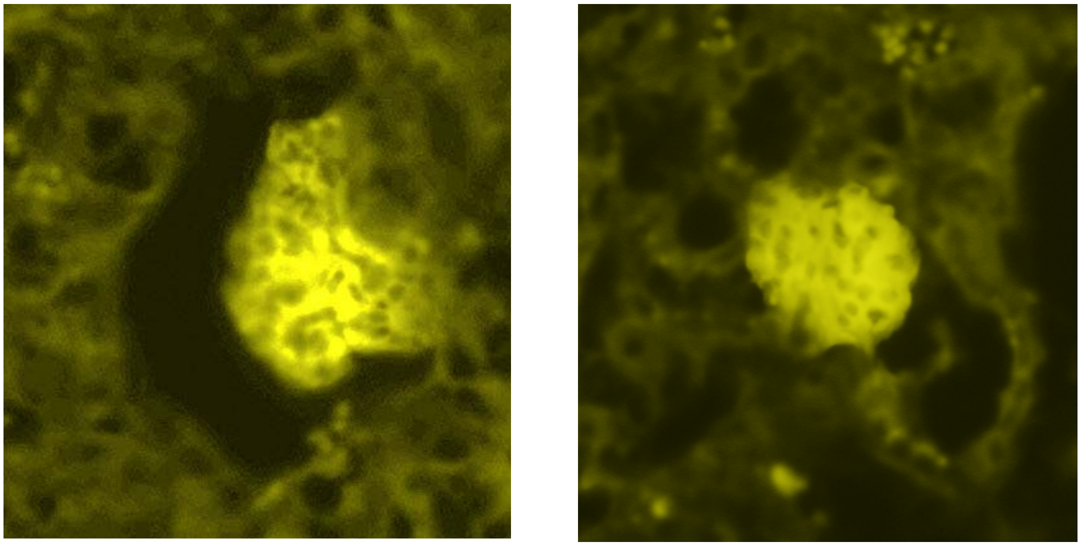

Supplementary Figure S7

Ex vivo and in vivo cytotoxicity test of anti-myeloma drugs to *Mef*$^{-/-}$ *Rad50*$^{s/s}$ plasma cell neoplasms.

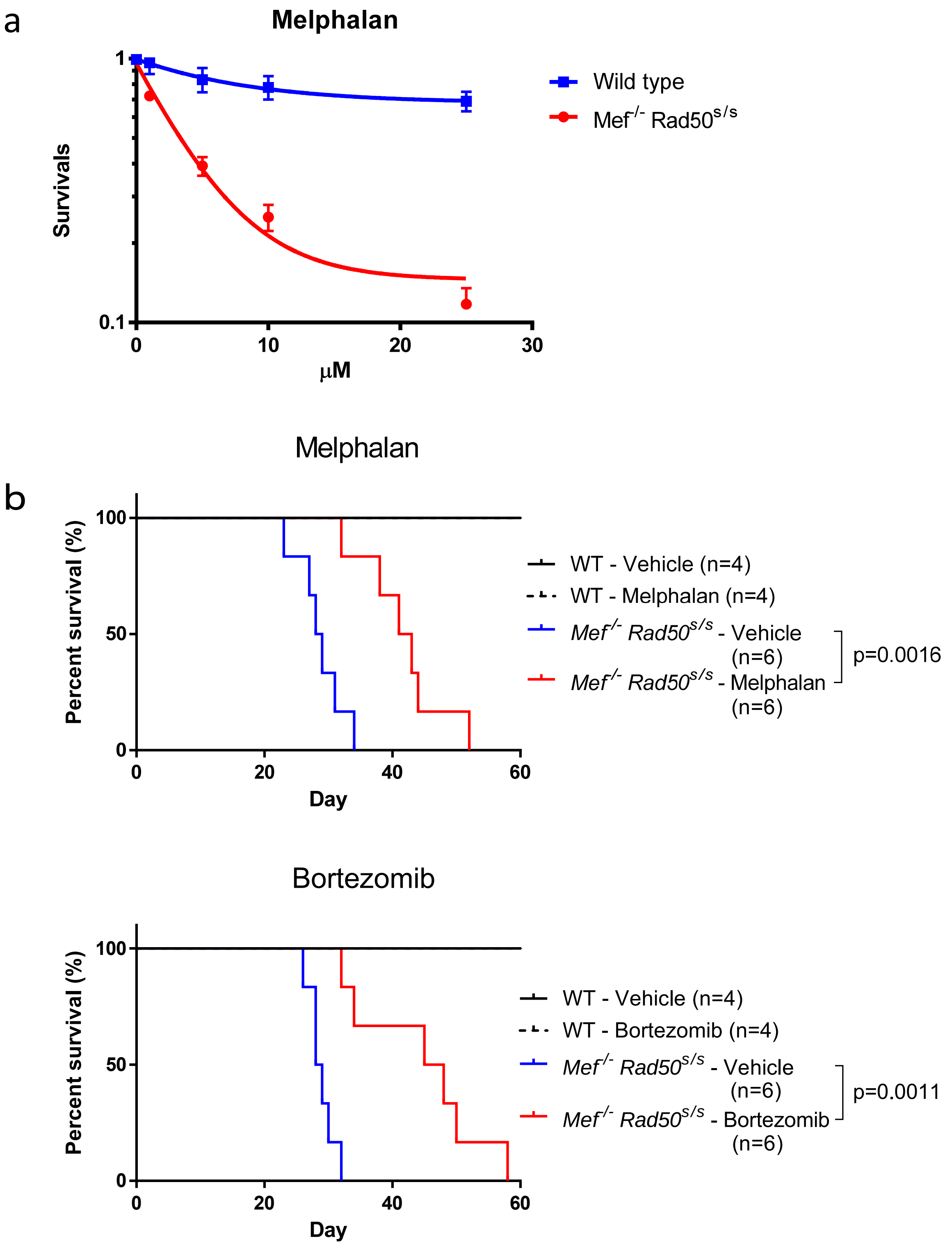

Supplementary Figure S8

Analysis of transcript expression levels of Bcl2 and Bax.

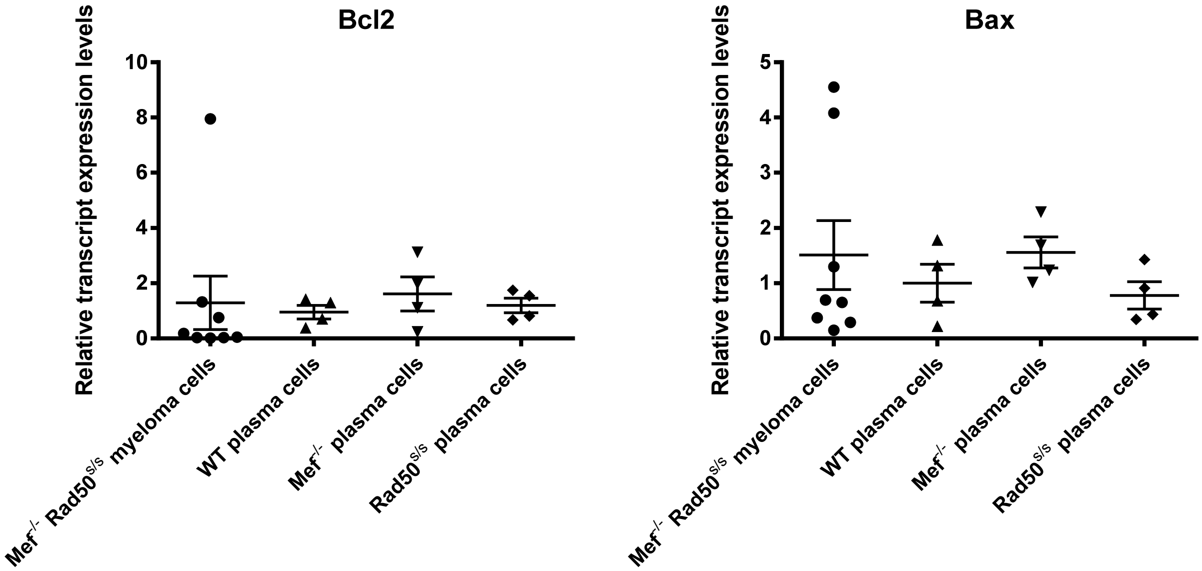

Supplementary Figure S9

Transcript expression level of some genes related to myelomagenesis.

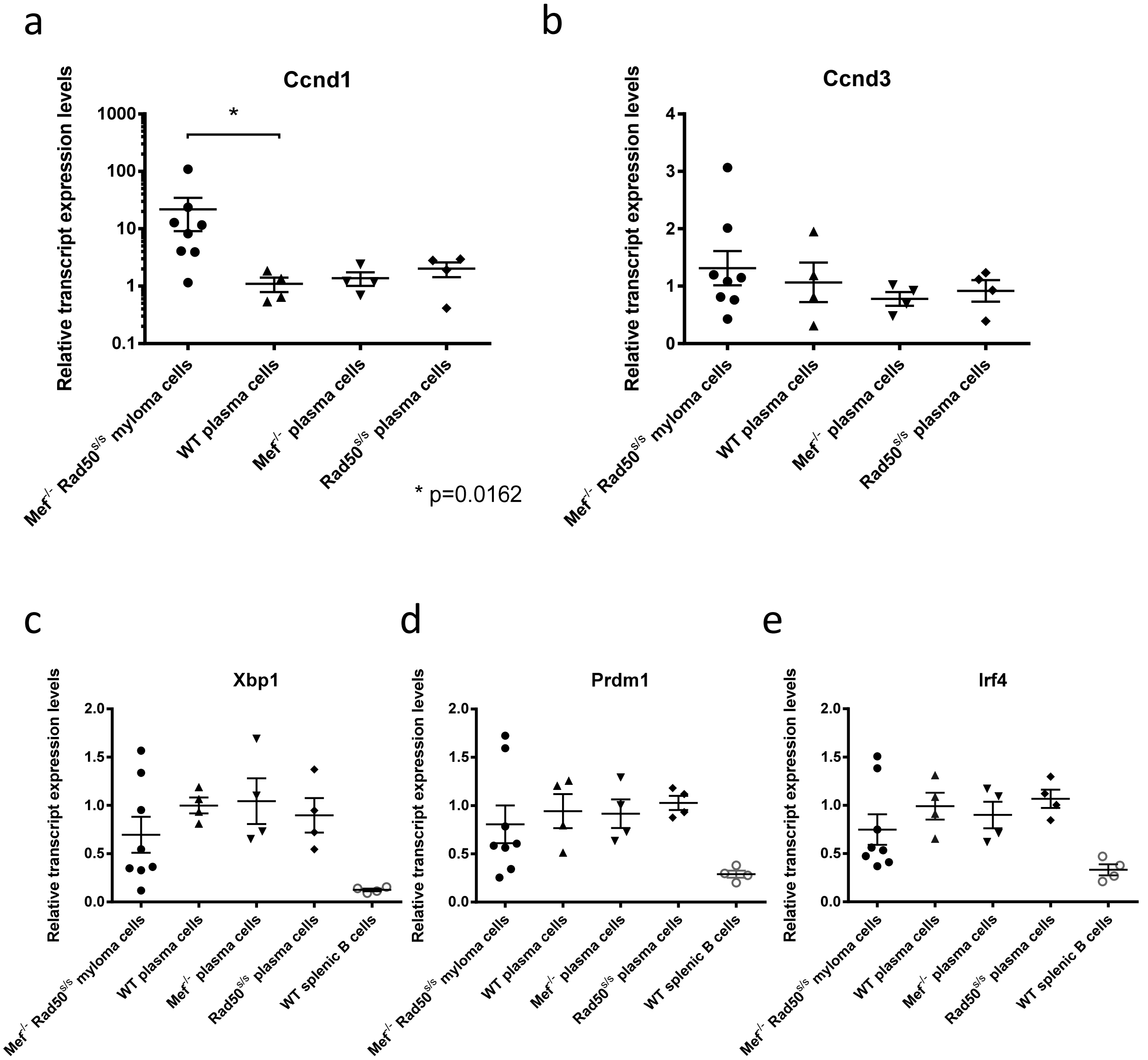

Supplementary Figure S10

Immunohistochemical analysis of Myc and CD138 in *Mef*$^{-/-}$ *Rad50*$^{s/s}$ tumors.

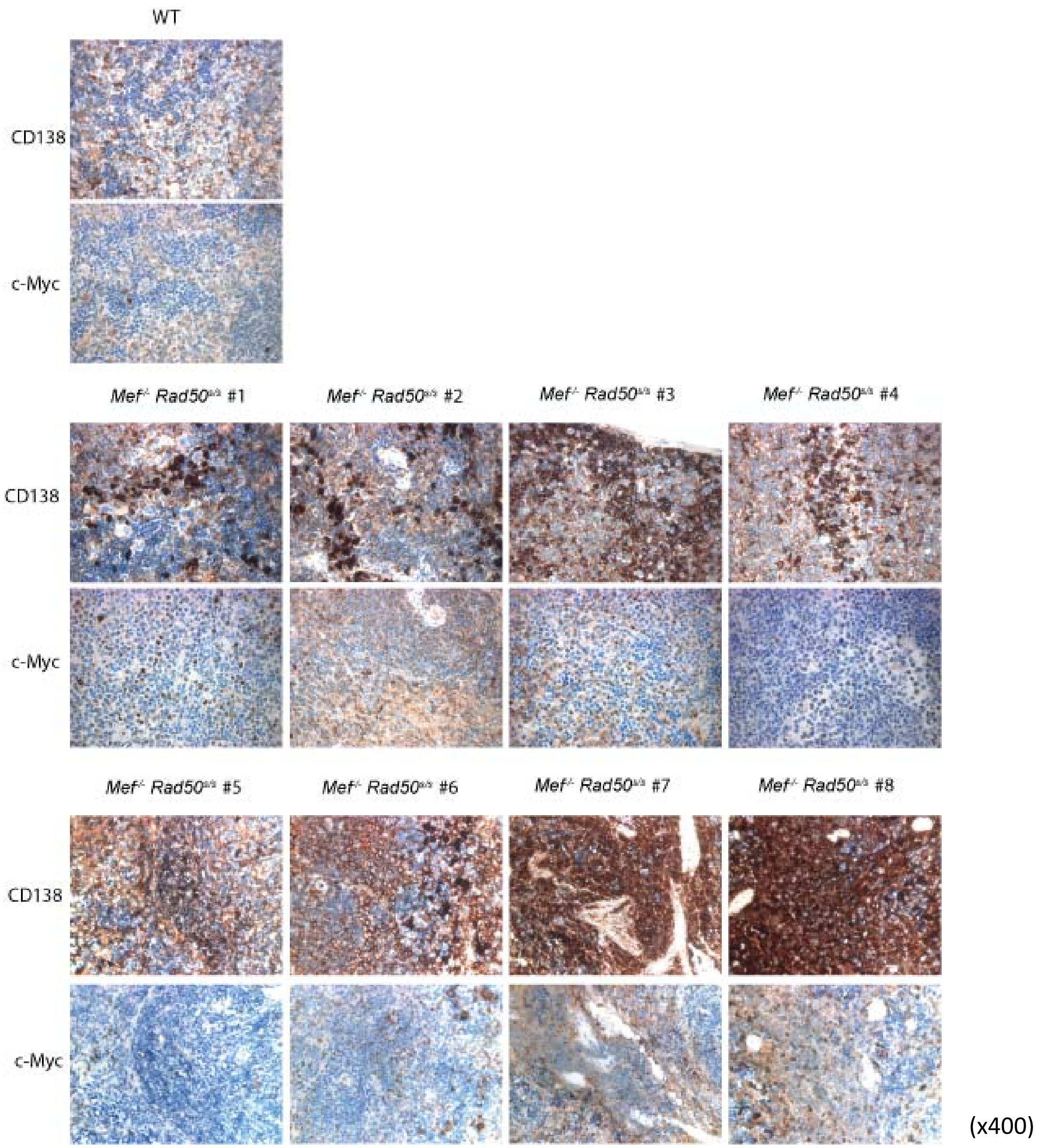

Supplementary Table S1

List of the *Mef*$^{-/-}$ *Rad50*$^{s/s}$ mice died over 200 days old.

Mice UID	Sex	Survival Days	Pathology	Ig class	FACS analysis (κ/λ)	PEL: M peak
1830	M	202	Bone marrow aplasia			
783	F	220	Multiple myeloma and plasmacytic infiltration to lung	IgG	κ	
784	F	220	Plasmacytosis in medullar of LNs and bone marrow	IgG	κ	
1304	F	231	Multiple myeloma in bone marrow BM and liver plasmacytosis			
2194	F	234	Unknown			
1752	M	264	Unknown			
2154	F	275	Multiple myeloma	not detected		
226	F	278	Unknown	N/A		
408	F	293	Multiple Myeloma with excess (>60%) of plasma cells in bone marrow	IgG	λ	
897	M	305	Multiple Myeloma	IgG	κ	
683	M	311	Plasma cell proliferation in bone marrow and spleen	not detected	κ	-
395	F	338	Multiple myeloma	IgG	κ	
776	M	345	Plasma cell hyperplasia in bone marrow	IgG	κ	
779	M	345	Plasmacytic leukemia	IgG	λ	
386	F	351	Multiple Myeloma with excess (>80%) of plasma cells in bone marrow	IgG	κ	
487	F	366	Unknown			
1341	F	379	Extramedullary plasmacytome			-
381	F	380	Plasma cell leukemia	IgG	κ	+
399	F	389	Extramedullary Plasmacytome, Lymphoplasmacytic inflammation in bone marow with Lymphoid depletion	IgG	κ	
561	M	416	Plasma cell hyperplasia in bone marrow		κ	-
609	M	428	Mediastinal plasmacytoma			+
116	M	456	Plasma cell proliferation in bone marrow	IgA	κ	+
833	F	478	Plasmacytic Luekimia	IgG	κ	+
328	F	494	Multiple myeloma			
1686	F	503	Multiple myeloma	IgG	λ	+
60	M	506	Multiple myeloma with plasmacytome	IgG	λ	
319	F	521	Multiple myeloma			+
112	F	566	Extramedullary plasmacytome	IgG	κ	
707	F	584	Multiple myeloma			+
396	F	607	Multiple Myeloma with pleural effusion	IgG	κ	+

Supplementary Table S2

List of the somatically mutated genes and mutations, which were identified in the list of somatic mutation observed in human multiple myeloma patients by the whole genome sequencing.

GENE NAME	SAMPLE	CHROMOSOME	REFERENCE	ALTERATION	MUTATION EFFECT	ENSAMBL TRANSCRIPT ID	AMINO ACID CHANGE	MUTATION FREQUENCY
Adam17	#3	chr12	A	C	NON_SYNONYMOUS_CODING	ENSMUST00000064536	L496R	0.5
Aff1	#3	chr5	C	A	NON_SYNONYMOUS_CODING	ENSMUST00000054979	P1218T	1
Agbl4	#3	chr4	C	T	STOP_GAINED	ENSMUST00000097920	E491*	0.375
Agbl4	#3	chr4	G	A	NON_SYNONYMOUS_CODING	ENSMUST00000097920	L493I	0.3333
Agbl4	#3	chr4	A	T	NON_SYNONYMOUS_CODING	ENSMUST00000097920	I497F	0.3704
Agbl4	#3	chr4	A	T	NON_SYNONYMOUS_CODING	ENSMUST00000097920	Q498H	0.4194
Agbl4	#3	chr4	C	A	STOP_GAINED	ENSMUST00000097920	W523*	0.4167
Agbl4	#3	chr4	A	T	NON_SYNONYMOUS_CODING	ENSMUST00000097920	N524Y	0.4225
Agbl4	#3	chr4	G	A	NON_SYNONYMOUS_CODING	ENSMUST00000097920	L533H	0.4167
Agbl4	#3	chr4	T	C	STOP_LOST	ENSMUST00000097920	*541C	0.4286
Aloxe3	#1	chr11	A	C	NON_SYNONYMOUS_CODING	ENSMUST00000021268	T312P	0.4286
Arhgef10	#4	chr8	G	C	NON_SYNONYMOUS_CODING	ENSMUST00000110800	G309R	0.3158
Arhgef10	#4	chr8	T	G	NON_SYNONYMOUS_CODING	ENSMUST00000110800	S1201A	0.25
Arhgef10	#4	chr8	G	A	NON_SYNONYMOUS_CODING	ENSMUST00000110800	V1202I	0.2759
Arhgef10	#4	chr8	A	G	NON_SYNONYMOUS_CODING	ENSMUST00000110800	T1212A	0.3478
Arhgef10	#4	chr8	A	C	NON_SYNONYMOUS_CODING	ENSMUST00000110800	N1225T	0.4211
Arhgef10	#4	chr8	G	A	NON_SYNONYMOUS_CODING	ENSMUST00000110800	R1247K	0.3913
Atp1a4	#1	chr1	G	A	NON_SYNONYMOUS_CODING	ENSMUST00000111243	E8V	1
Cacna1c	#3	chr6	A	G	NON_SYNONYMOUS_CODING	ENSMUST00000112825	L1664S	1
Camta1	#1	chr4	A	G	NON_SYNONYMOUS_CODING	ENSMUST00000049790	L336P	1
Ccdc123	#3	chr7	C	G	NON_SYNONYMOUS_CODING	ENSMUST00000079414	A58G	1
Cftr	#2	chr6	C	G	NON_SYNONYMOUS_CODING	ENSMUST00000045706	S13A	0.5833
Chd3	#4	chr11	T	A	STOP_GAINED	ENSMUST00000092971	Q1004*	0.8333
Cpsf3	#3	chr12	T	C	NON_SYNONYMOUS_CODING	ENSMUST00000067284	T402P	0.3958
Csmd1	#4	chr8	C	T	NON_SYNONYMOUS_CODING	ENSMUST00000082104	R3394Q	0.6
Csmd1	#4	chr8	C	T	NON_SYNONYMOUS_CODING	ENSMUST00000082104	S2844N	0.4048
Csmd1	#4	chr8	G	T	NON_SYNONYMOUS_CODING	ENSMUST00000082104	T2829K	0.4706
Csmd1	#4	chr8	G	T	NON_SYNONYMOUS_CODING	ENSMUST00000082104	T1148N	0.4667
Csmd1	#4	chr8	T	C	NON_SYNONYMOUS_CODING	ENSMUST00000082104	I345V	0.3793
Cux2	#3	chr5	T	C	NON_SYNONYMOUS_CODING	ENSMUST00000086317	L521R	0.9
Dock10	#1	chr1	A	G	NON_SYNONYMOUS_CODING	ENSMUST00000077946	E1697A	1
Dock10	#1	chr1	G	T	NON_SYNONYMOUS_CODING	ENSMUST00000077946	I222N	1
Donson	#4	chr16	G	A	NON_SYNONYMOUS_CODING	ENSMUST00000023682	Q211H	0.7333
Dspp	#1	chr5	A	G	NON_SYNONYMOUS_CODING	ENSMUST00000112771	D855G	0.8333
Gabrb2	#2	chr11	T	G	NON_SYNONYMOUS_CODING	ENSMUST00000007797	D450E	1
Gas7	#1	chr11	A	G	NON_SYNONYMOUS_CODING	ENSMUST00000041611	S51A	1
Gpatch2	#1	chr1	C	G	NON_SYNONYMOUS_CODING	ENSMUST00000160471	S241G	0.5714
Ift122	#2	chr6	C	G	NON_SYNONYMOUS_CODING	ENSMUST00000038234	Y235D	1
Ift122	#2	chr6	A	G	NON_SYNONYMOUS_CODING	ENSMUST00000038234	M551V	1
Ift122	#2	chr6	C	T	NON_SYNONYMOUS_CODING	ENSMUST00000038234	L691F	1
Kcnc1	#3	chr7	A	G	NON_SYNONYMOUS_CODING	ENSMUST00000160433	E541G	1
Kif1a	#4	chr1	T	C	NON_SYNONYMOUS_CODING	ENSMUST00000086819	N90K	0.7143
Ksr2	#3	chr5	G	C	NON_SYNONYMOUS_CODING	ENSMUST00000180430	K283T	1
Lrp8	#3	chr4	G	A	NON_SYNONYMOUS_CODING	ENSMUST00000126573	C586Y	0.5
Lrp8	#3	chr4	T	C	NON_SYNONYMOUS_CODING	ENSMUST00000126573	N653T	0.7
Mcf2l	#4	chr8	A	G	NON_SYNONYMOUS_CODING	ENSMUST00000145067	T8A	0.4865
Mcf2l	#4	chr8	A	G	NON_SYNONYMOUS_CODING	ENSMUST00000145067	S9G	0.4789
Mcf2l	#4	chr8	G	T	STOP_LOST	ENSMUST00000110867	*1102L	0.4286
Mphosph9	#3	chr5	A	G	NON_SYNONYMOUS_CODING	ENSMUST00000031344	S934R	1
Mphosph9	#4	chr5	A	G	NON_SYNONYMOUS_CODING	ENSMUST00000031344	S934R	0.9444
Mphosph9	#3	chr5	TA	T	FRAME_SHIFT	ENSMUST00000031344	-163	0.5455
Mpl	#3	chr4	C	T	NON_SYNONYMOUS_CODING	ENSMUST00000106375	S243N	0.5254
Mpl	#3	chr4	G	A	STOP_GAINED	ENSMUST00000106375	Q238*	0.4571
Myo5a	#1	chr9	A	AG	FRAME_SHIFT	ENSMUST00000123128	-681?	1

Supplementary Table S2(Cont.)

Myom2	#4	chr8	G	A	NON_SYNONYMOUS_CODING	ENSMUST00000033842	R123Q	0.4107
Myom2	#4	chr8	A	C	NON_SYNONYMOUS_CODING	ENSMUST00000033842	M131L	0.3433
Myom2	#4	chr8	T	C	NON_SYNONYMOUS_CODING	ENSMUST00000033842	S234P	0.4545
Myom2	#4	chr8	C	A	NON_SYNONYMOUS_CODING	ENSMUST00000033842	L977I	0.3676
Myom2	#4	chr8	G	A	NON_SYNONYMOUS_CODING	ENSMUST00000033842	V1182I	0.4783
Myom2	#4	chr8	G	A	NON_SYNONYMOUS_CODING	ENSMUST00000033842	S1311N	0.4048
Nefm	#1	chr14	G	A	NON_SYNONYMOUS_CODING	ENSMUST00000022638	T408I	0.7143
Nfia	#1	chr4	A	AT	FRAME_SHIFT	ENSMUST00000052018	-16?	1
Numa1	#3	chr7	A	C	NON_SYNONYMOUS_CODING	ENSMUST00000084852	I848L	1
Obscn	#1	chr11	A	G	NON_SYNONYMOUS_CODING	ENSMUST00000047441	D7634A	1
Obscn	#1	chr11	C	T	NON_SYNONYMOUS_CODING	ENSMUST00000047441	Y7632N	1
Obscn	#4	chr11	T	C	NON_SYNONYMOUS_CODING	ENSMUST00000047441	I7317V	1
Obscn	#3	chr11	A	G	NON_SYNONYMOUS_CODING	ENSMUST00000047441	V7293L	1
Obscn	#1	chr11	A	C	NON_SYNONYMOUS_CODING	ENSMUST00000047441	V7208G	1
Obscn	#4	chr11	AC	A	FRAME_SHIFT	ENSMUST00000047441	-6833	1
Obscn	#4	chr11	A	G	NON_SYNONYMOUS_CODING	ENSMUST00000047441	S6597T	1
Obscn	#3	chr11	C	T	NON_SYNONYMOUS_CODING	ENSMUST00000047441	E6476K	1
Obscn	#4	chr11	G	T	NON_SYNONYMOUS_CODING	ENSMUST00000081658	A1127T	1
Obscn	#1	chr11	C	T	NON_SYNONYMOUS_CODING	ENSMUST00000047441	S3626N	1
Pfas	#4	chr11	C	T	NON_SYNONYMOUS_CODING	ENSMUST00000021282	F220L	1
Pkhd1	#1	chr1	G	A	NON_SYNONYMOUS_CODING	ENSMUST00000088448	L1872F	0.8333
Pkhd1l1	#2	chr15	AGT	A	FRAME_SHIFT	ENSMUST00000038336	-837	0.3158
Prkcz	#1	chr4	A	G	NON_SYNONYMOUS_CODING	ENSMUST00000103178	G230A	0.8889
Prkd2	#4	chr7	G	A	NON_SYNONYMOUS_CODING	ENSMUST00000086104	V121E	1
Ptk2	#2	chr15	G	A	STOP_GAINED	ENSMUST00000110036	K381*	1
Ptk2	#2	chr15	T	C	SPLICE_SITE_ACCEPTOR	ENSMUST00000110036	.	1
RALYL	#3	chr3	A	G	NON_SYNONYMOUS_CODING	ENSMUST00000108373	I10V	0.3429
Rasgrf1	#3	chr9	CA	C	FRAME_SHIFT	ENSMUST00000034912	-944	0.5556
Ripk4	#4	chr16	T	G	NON_SYNONYMOUS_CODING	ENSMUST00000113743	L528P	1
Ripk4	#4	chr16	A	G	NON_SYNONYMOUS_CODING	ENSMUST00000113743	W518C	1
Ripk4	#4	chr16	G	A	NON_SYNONYMOUS_CODING	ENSMUST00000113743	A517V	1
Serpine2	#1	chr1	G	A	NON_SYNONYMOUS_CODING	ENSMUST00000027467	M219I	1
Serpine2	#1	chr1	T	C	NON_SYNONYMOUS_CODING	ENSMUST00000027467	Q216E	1
Slc1a5	#4	chr7	C	T	NON_SYNONYMOUS_CODING	ENSMUST00000108496	S217L	0.9
Slc1a5	#4	chr7	T	C	NON_SYNONYMOUS_CODING	ENSMUST00000108496	L469S	0.8333
Slc1a5	#4	chr7	A	G	NON_SYNONYMOUS_CODING	ENSMUST00000108496	L471V	0.8333
Spta1	#1	chr1	C	T	NON_SYNONYMOUS_CODING	ENSMUST00000027817	C222F	0.8333
Spta1	#1	chr1	A	G	NON_SYNONYMOUS_CODING	ENSMUST00000027817	S1127R	0.7
Spta1	#3	chr1	C	T	NON_SYNONYMOUS_CODING	ENSMUST00000027817	K1439I	0.5385
Spta1	#1	chr1	A	G	NON_SYNONYMOUS_CODING	ENSMUST00000027817	V2406G	0.5556
Srrm2	#2	chr17	C	A	NON_SYNONYMOUS_CODING	ENSMUST00000088621	A542D	1
Sytl2	#1	chr7	C	T	NON_SYNONYMOUS_CODING	ENSMUST00000107210	P168L	1
Tada2a	#3	chr11	A	G	NON_SYNONYMOUS_CODING	ENSMUST00000018795	N120H	1
Tecpr1	#1	chr5	C	T	NON_SYNONYMOUS_CODING	ENSMUST00000085701	L714I	1
Tmcc1	#2	chr6	TAGGA	T	FRAME_SHIFT	ENSMUST00000088896	-101	1
Vps13d	#1	chr4	G	A	NON_SYNONYMOUS_CODING	ENSMUST00000020441	E1551D	0.625
Vps13d	#1	chr4	G	T	NON_SYNONYMOUS_CODING	ENSMUST00000020441	I1400K	1
Vps13d	#1	chr4	C	T	STOP_GAINED	ENSMUST00000020441	L1393*	0.8333
Zan	#3	chr5	T	A	STOP_GAINED	ENSMUST00000117564	K4109*	1
Zan	#3	chr5	T	G	NON_SYNONYMOUS_CODING	ENSMUST00000117564	D2883H	1
Zkscan3	#1	chr13	C	A	STOP_GAINED	ENSMUST00000070785	Q426*	0.4043

Supplementary Table S3

Functional Annotation Clustering by David Software using the gene mutations which we found in *Mef*$^{-/-}$ *Rad50*$^{s/s}$ plasma cell neoplasms.

Clustering	P Value	Gene No.	Gene Names
ABC transporter	0.023	5	Abca5, Abca9, Abcc3, Abcd1, Cftr
NF-KB signaling	0.026	6	Relb, Nfat5, Nfkb1, Camta1, Pkhd1, Pkhd1l1
Notch signaling	0.032	5	Notch2, Adam17, Dll3, Ncstn, Psen2
Focal adhesion	0.005	10	Ptk2, Rasgrf1, Rock1, Rock2, Chad, Col4a1, Col5a3, Lama3, Myl2, Prkca

Supplementary Table S4

Comparison of the biological and clinical features of multiple myeloma mouse models.

	EμXBP1	Vκ*MYC	*Mef*$^{-/-}$ *Rad50*$^{s/s}$	Human Multiple Mueloma
Median survival	more than 700 days	661 days	478 days	
Diagnosis of plasma cell tumors	26% @ 44-80w	70% @ 80w	70% @ 44w	
Location	BM, EM	BM, EM	BM, EM	BM
Slowly progressive	+	+	+	+
SHM (median)	No	2.6%	3.2%	6-8 %
Most common Ig subtyoe	IgM > IgG	IgG	IgG	IgG
Age-related M-spike	Yes 30% @ 50w	Yes 80% @ 50w	Yes 73% @ 44w	Yes
Transplantavility	n.d	+	+	+ to SCID mice
Anemia	n.d	Yes	Yes	Yes
Osteoporosis	n.d	Yes	Yes	Yes
Kidney disease	Yes	Yes	Yes	Yes
Myc overexpression	n.d	100%	63%	67%
